# Novel cobalt–carbon@silica adsorbent

**DOI:** 10.1038/s41598-020-75367-0

**Published:** 2020-10-29

**Authors:** Nusaybah Alotaibi, Hassan H. Hammud, Nasreen Al Otaibi, Syed Ghazanfar Hussain, Thirumurugan Prakasam

**Affiliations:** 1 Department of Chemistry, College of Science, King Faisal University, P.O Box 400, Al-Ahsa, 31982 Saudi Arabia; 2 Department of Physics, College of Science, King Faisal University, P.O Box 400, Al-Ahsa, 31982 Saudi Arabia; 3 Chemistry Program, New York University Abu Dhabi (NYUAD), Abu Dhabi, United Arab Emirates

**Keywords:** Environmental sciences, Chemistry, Materials science, Nanoscience and technology

## Abstract

Recently, carbon nanostructures are of high importance due to their unique characteristics and interesting applications. Pyrolysis of anthracene with cobalt complex Co(2,2′-bipy)Cl_2_ **(1)**, where (2,2′-bipy) is 2,2′-bipyridine, in the absence and presence of silica gave in high yield cobalt-carbon nanocomposite CoCNC **(2)** and CoCNC@SiO_2_ **(3)** at 600 °C and 850 °C, respectively. They were characterized using SEM, TEM, PXRD, Raman and XPS. **(3)** and **(2)** contain core–shell cobalt(0)/cobalt oxide-graphite with or without silica support. PXRD indicates that **(2)** contains crystalline hexagonal α-Co and cubic β-Co phases while **(3)** contains only cubic β-Co phase and silica. The structure of **(2)** is 3D hierarchical carbon architecture wrapping spherical and elliptical cobalt nanoparticles. **(3)** consists of graphitized structures around cobalt nanoparticles embedded in the silica matrix. XPS reveals that the nanocomposites contain oxygen functional groups that enhance uptake of cationic dyes. CoCNC@SiO_2_ **(3)** has higher capacity and thus is better adsorbent of Basic Violet 3 than CoCNC **(2)**. The Langmuir adsorption capacity of **(3)** is 19.4 mg g^−1^ while column capacity is 12.55 mg g^−1^ at 25 °C. Freundlich isotherm and pseudo-second-order kinetic models fit well the adsorption data. Thermodynamics indicate that adsorption**(3)** is exothermic. Column regeneration was tested for three cycles and Yan et al. was found the best kinetic model.

## Introduction

Recently, environmental pollution by dyes has attracted considerable attention. Many dye industries discharge a considerable amount of colored water that hinders sunlight penetration which is crucial for photosynthesis in aquatic ecosystems^1,2^. In addition, toxic dyes cause health problems to humans, such as respiratory irritation and water-borne diseases^3^, hence, many techniques have been explored in order to remove toxic dyes from water, such as coagulation, flocculation^4^, filtration^5^, biodegradation^6^ and electrochemical decolorization^7^. Among dye removal methods, adsorption process has been one of the most effective alternatives due to different factors, such as simplicity, low cost and feasibility^8^. Besides, it does not involve production of toxic biproducts^3^ that requires post treatment.

Many dye adsorbents have been explored including activated carbon, which has some disadvantages that limit its use as an effective adsorbent, such as high cost as the high energy requirement and carbonization time can make the activation process expensive. In addition, the carbonization temperature and time depend significantly on the feedstocks, a series of pre-activation steps is required and chemical activation requires impregnation before heat treatment under a controlled environment which make the generation process complicated^9,10^. Hence, new adsorbents have been prepared to overcome the limitations associated with conventional adsorbents. One of the most widely used is carbon nanotubes, which have been proven for its efficiency toward industrial dyes removal due to its remarkable properties, such as high surface area and thermal and chemical stability^11,12^. The efficient adsorption behavior of carbon nanotubes paves the way for preparing other nanomaterials, such as metal–carbon nanocomposites, which have recognizable adsorption capacity toward a wide range of dyes since encapsulating metal particles decreases diffusion resistance and promote mass transfer^13^. In addition, it provides new features such as magnetism^14^ and specific binding sites can be created^13–15^. Another benefit is that they can be easily removed from the aqueous solution after adsorption by applying an external magnetic field instead of centrifugation or filtration^13^.

Many techniques have been used in order to prepare efficient carbon nanomaterials, including arc discharge^16^, laser ablation^17^ and chemical vapor deposition^18^. Although these methods provide high-quality nanotubes, they are not economically advantageous which limits their use in large-scale production. The previous methods are expensive in terms of instruments and energy consumption. Arc discharge requires high temperature (above 1700 °C) and the temperature requires for laser ablation and chemical vapor deposition can reach > 1100 °C^10,19^. In addition, chemical vapor deposition requires expensive metal nanoparticles as catalysts. Therefore, other low-cost synthetic approaches have been explored to meet the substantial demand of highly effective carbon nanomaterials, such as pyrolysis of organometallic complexes. In fact, it is found that organometallic complexes decrease the temperature required for carbonization compared to metal-free organic molecules. In addition, different carbon nanostructures can be formed and controlled by choosing the appropriate precursors and optimizing pyrolysis temperatures^20–22^. Therefore, the solid-state pyrolysis of organometallic complexes is a recognizable alternative for preparing carbon nanostructures with a broad range of sizes and shapes under optimized conditions. It affords metal nanoparticles catalyst for graphitization at low temperature, while carbon gas generated from decomposed organic ligand diffuses and deposits on metal nanoparticles upon saturation to produce the desired carbon nanostructures. Several researchers have reported the pyrolytic synthesis of carbon nanostructures using organometallic compounds. For instance, Fe-filled carbon nanotubes CNTs were obtained via solid-state pyrolysis of ferrocene at 645 °C^23^ and butadiynyl-ferrocene-containing compounds at 1300 °C^24^.

Nickel-encapsulated CNTs have also been obtained after pyrolysis of nickel complex with benzene-1,3,5-tricarboxylato at 500 °C for 20 h^22^. Besides Fe and Ni, Co has been proven for its efficient catalytic behavior in solid-state pyrolysis approach. It is found that the pyrolysis of cobalt complex with hexa-*peri*-hexabenzocoronene results in the formation of uniform straight CNTs after gradual heating up to 1000 °C^25^. Other carbon nanostructures, such as carbon/cobalt nanorods, and carbon/cobalt nanospheres can be prepared via pyrolysis of polyphenylene dendrimer/cobalt complexes at high temperature up to 800 °C^26^.

Although, several metal–carbon nanocomposites are reported and tested as dye adsorbents^27–30^. There is still a need to further investigate the adsorption behavior of the new materials prepared using a solid-state novel approach. In addition, Basic Violet 3 dye has been chosen as target adsorbate because of different factors. It has been used for medication as biological stain, mutagenic and bacteriostatic agent and fungal growth inhibitors. In addition, it is used extensively as dyeing industries, such as textile dyeing, paints and painting ink^31^. Despite its wide range uses, Basic Violet 3 molecules exhibit high persistence in the environment as well as have toxic effects, such as severe kidney failure^32^, skin irritation, pulmonary disease and cancer in humans^33^. It can be fatal or causes mutations in organisms^34^ which makes it classified as a biohazardous material^31^. Herein, mesoporous cobalt-carbon nanostructure is obtained by pyrolysis of cobalt 2,2′-bipyridine complex with additional carbon source anthracene and support silica. 2,2′-bipyridine being a nitrogen-aromatic ligand, can play an important role in preparation of graphitized nitrogen-doped carbon. The presented method is considered to be economically suitable, since in addition to the easy prepared cobalt 2,2′- bipyridine complex, cheap materials anthracene and silica were used each as 1/3 of the starting materials. The temperature used was also relatively low (600, 850 °C). Therefore, highly porous metal/carbon nanocomposites can be produced at large scale. In addition, silica support makes **(3)** to be easily packed into a column for industrial application in water treatment.

## Materials and methods

### Materials

Cobalt(II) chloride hexahydrate (98%, Panreact), 2,2′-bipyridine (99.5%, Loba Chemie), ethanol (ACS grade, Scharlau), hydrochloric acid (37%, Panreact), Basic Violet 3 (colour index 42,555, Acros Organics). Silica gel 60 (0.063–0.10 mm) (Merck).

### Synthesis of Co(2,2′-bipy)Cl_2_ (1)

Co(2,2′-bipy)Cl_2_ **(1)** complex was prepared using literature procedure^35^; Cobalt chloride hexahydrate (1.832 g, 7.7 mmol) in 30 mL ethanol was added to another solution of 2,2′-bipyridine (0.875 g, 5.6 mmol) in ethanol (30 mL). Within a few minutes, a blue precipitate formed, the reaction mixture was refluxed for 4 h. The blue precipitate was collected by filtration, washed with ethanol and allowed to air dry, resulting in 1.75 g yield (79.46% based on cobalt). Analysis (%) Calc. for CoCl_2_C_10_H_8_N_2_: C, 41.99; H, 2.82; N, 9.79. Found: C, 41.9; H, 2.58; N, 10.15. FTIR and thermal analysis are presented and discussed in the supplementary (Table S1, Figs. S1 and S2).

### Synthesis of cobalt-carbon nanocomposite CoCNC (2) and CoCNC@SiO_2_ (3)

CoCNC **(2)** was prepared by mixing Co(2,2′-bipy)Cl_2_ **(1)** (0.35 g) and anthracene (0.35 g). Then, the mixture was transferred to a crucible with a lid and heated in a furnace under increased and constant temperature for different time intervals up to 600 °C under vacuum/nitrogen atmosphere. After a slow cooling to room temperature, a black powder of **(2)** was obtained (0.2 g).

CoCNC@SiO_2_ **(3)** was prepared similarly by mixing Co(2,2′-bipy)Cl_2_ **(1)** (1 g), anthracene (1 g) and silica (1 g). Then, the mixture was heated under increased and constant temperature for different time intervals up to 850 °C under a vacuum/nitrogen atmosphere. After a slow cooling to room temperature, a black powder of **(3)** was obtained (1.85 g). An illustrative diagram of the cobalt-carbon nanocomposite synthesis is shown in Fig. S3.

### Characterization techniques

Morphological characterization was achieved using Scanning Electron Microscope (FE-SEM, QuantaFEG450, FEI) and (FESEM, JSM-6460LV). Edax elemental mapping and elemental composition were obtained using dual beam scanning electron microscope (ThermoFisher Scios). Transmission electron microscopy (TEM) images were obtained using (JEOL-JEM-1011, Tokyo, Japan) operated at 80 kV. X-ray Powder Diffraction (XRD) was carried out using Inel Equinox 1000 powder diffractometer supplied with a CPS 180 detector (filtered Co Kα1 irradiation, 30 kV, 30 mA, λ = 1.789 Å, zero background spinning sample holder). Powder pattern was analyzed using Mass Crystal Impact software (v.1.11e) for phase identification, both COD and ICSD databases were used. Raman spectra were measured using (DXR, Thermo scientific). X-ray Photoelectron Spectroscopy (XPS) measurements were carried out on SPECS GmbH high vacuum multi-technique surface analysis system supplied with an Mg-Kα 1253.6 eV X-ray source. Calibration of the spectra was achieved by setting C 1*s* line at 284.8 eV. FTIR spectra were measured on Fourier transform infrared spectroscopy (Cary 630 FTIR Spectrometer, Agilent) instrument. Elemental analyses (CHN) were performed on a Perkin-Elmer 2400 elemental analyzer. The thermogravimetric analysis and differential thermal analysis (TGA-DTA) experiment was non-isothermal decomposition experiments. The experiments were run in an inert atmosphere using nitrogen as flow gas, which was introduced into the TGA furnace (SDT Q600 V20.9 Build 20). The heating rate used was of 3 °C min^−1^ and was maintained constant. The final temperature was set at 800 °C. The porosity of **(2)** and **(3)** were measured using N_2_ adsorption/desorption isotherms at 77 K after degassing the nanocarbon samples at 90 °C for 36 h to ensure pores free from trapped gas and solvent molecules (TriStar II Plus 2.02, Micromeritic).

### Adsorption study

In isotherm experiments, flasks containing 10 mL of dye solution with initial concentration C_o_ (50, 100, 150, 200, 250, 300 mg L^−1^) and 0.04 g of the adsorbent CoCNC@SiO_2_ **(3)** were shaken at 120 rpm rotation speed. While for kinetics experiment, 0.2 gm adsorbent in 50 ml dye solution (100 ppm) was used. The remaining dye concentration C_e_ (mg L^−1^) was obtained by reading the absorbance using UV/Vis spectrophotometer (Shimadzu, UV-1800) at λmax 582 nm. The quantity of dye adsorbed at equilibrium time, q_e_ (mg g^−1^), was then calculated. The pH of the solution was adjusted using dilute solution of NaOH or HCl. A fixed-bed column study was conducted using a column of 22 cm height and 1 cm inner diameter. The column was packed with 0.5 g of **(3)** and eluted with 50 ppm dye solution at 1 mL min^−1^ flow rate. After regeneration with dilute HCl, the column bed was washed with deionized water with continuous measurement of pH, to ensure the removal of excess H^+^ ions from the column before CoCNC@SiO_2_ **(3)** was reused for next adsorption cycles.

## Results and discussion

### Characterization of cobalt-carbon nanostructures

#### Morphological structure

**Figure 1:**
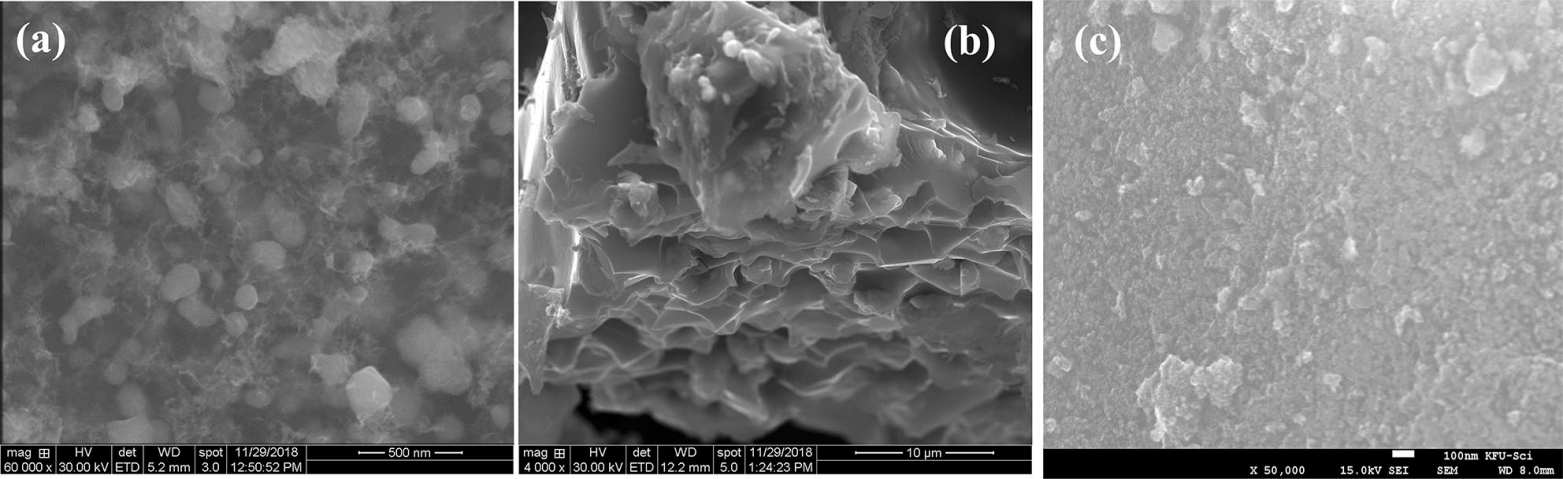
SEM images of pyrolytic products of the precursor Co(2,2′-bip)Cl_2_ at different pyrolysis conditions. (**a**) SEM image of CoCNC **(2)** shows 3D hierarchical carbon architecture decorated with spherical and elliptical cobalt nanoparticles. (**b**) cross-sectional image of CoCNC **(2)**. (**c**) SEM image of CoCNC@SiO_2_ **(3)** shows porous cobalt carbon nanostructures coated on large silica particles.

**Figure 2:**
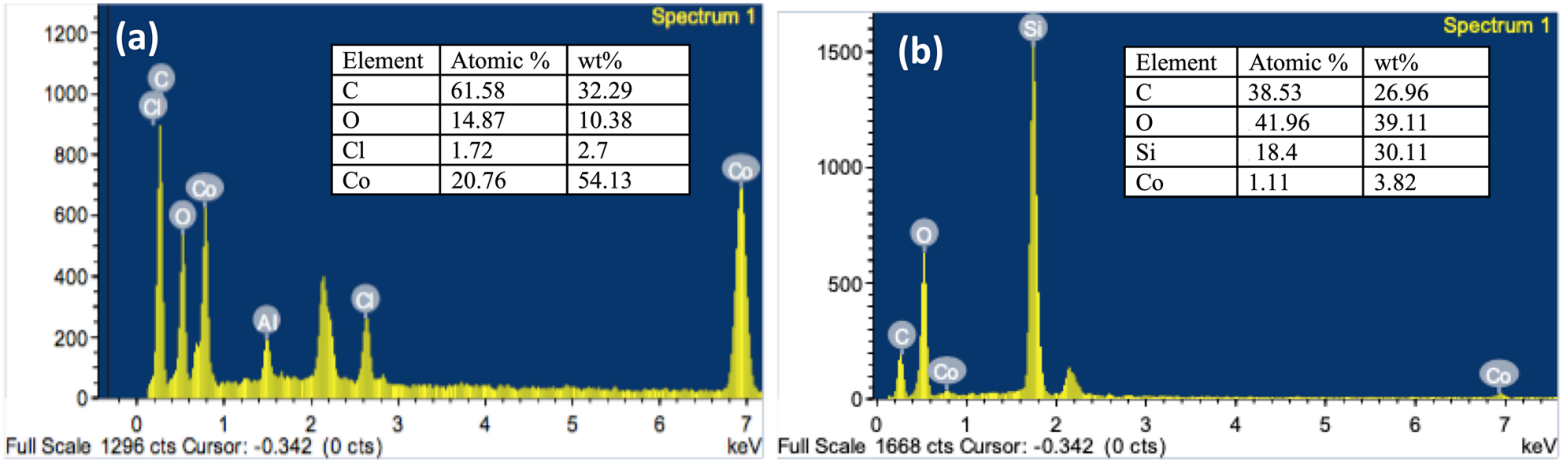
EDX spectra and weight percentages of detected elements. (**a**) CoCNC **(2)**. (**b**) CoCNC@SiO_2_ **(3)**. **(3)** shows a new peak of silicon and a noticeable decrease in % carbon and an increase in % of cobalt compared to **(2)**.

**Figure 3:**
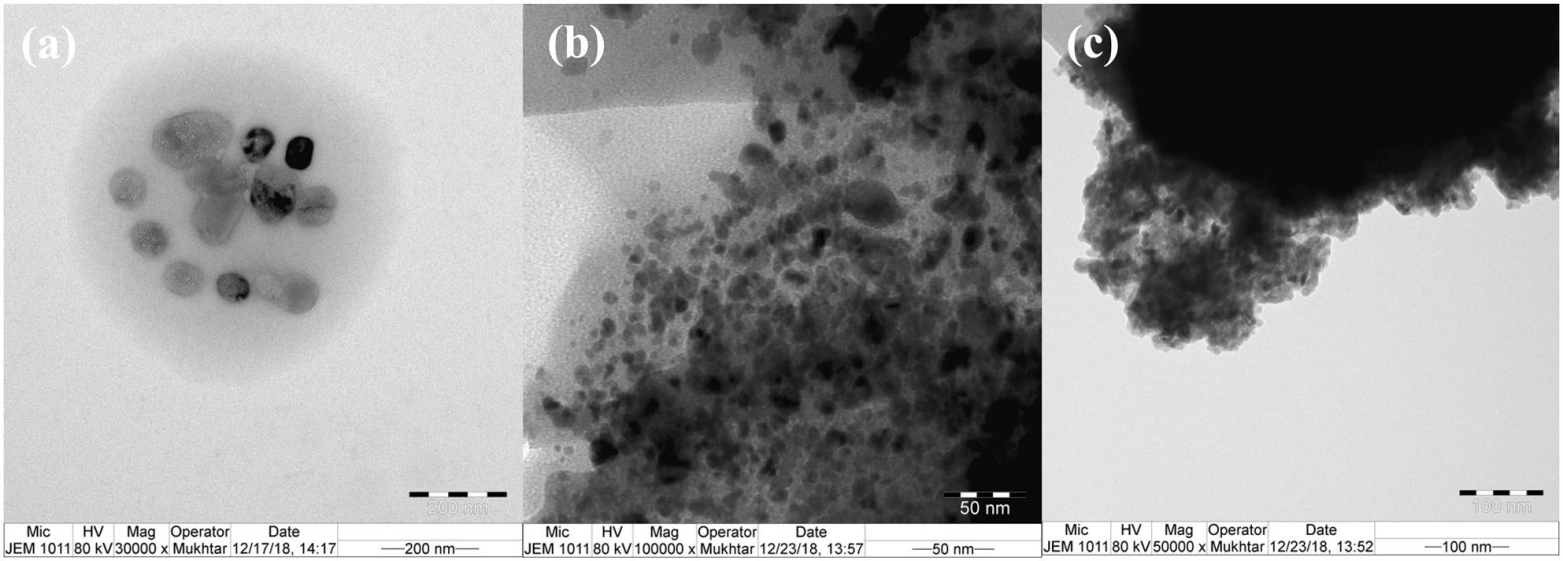
(**a**) TEM image of CoCNC **(2)** showing spherical cobalt nanoparticles. (**b**) and (**c**) TEM images of CoCNC@SiO_2_ **(3)** showing the graphitized structure around cobalt nanoparticles embedded in the silica matrix.

Figure 1 shows the SEM images of CoCNC **(2)**, the product of pyrolysis of Co(2,2′-bipy)Cl_2_ **(1)** with anthracene in 1:1 weight ratio at 600 °C for 10 h. The structure consists of porous 3D hierarchical carbon architecture wrapping white spherical and elliptical cobalt particles with an estimated size of < 100 nm. Furthermore, cross-sectional image at lower magnification (10 μm) shows macroporous channels of estimated pore diameter less than 10 μm and wall thickness less than 1 μm. The appearance of macroporous channels may be attributed to the randomly cross-linked graphene sheets^36^. EDX spectrum (Fig. 2) indicated the presence of oxygen in **(2)** due to adsorbed water or air^37^ of the freshly prepared cobalt-carbon nanoparticles. While the presence of chlorine is derived from the starting complex Co(2,2′-bipy)Cl_2_ **(1)**. This suggests the doping of chlorine in the obtained carbon nanostructures. In addition, pyrolysis at 850 °C for 15 h of complex, anthracene and silica in (1:1:1 ratio) resulted in highly porous cobalt carbon nanostructures coated on large silica particles CoCNC@SiO_2_ **(3)** as indicated by SEM image (Fig. 1), while TEM image of (**3**) (Fig. 3) shows graphitized carbon-cobalt (shell-core) particles embedded in the silica matrix. TEM image of **(2)** clearly shows spherical porous cobalt nanoparticles. EDX of **(3)** shows an oxygen peak derived from silica and adsorbed air and water. It shows a new huge peak of silicon and a noticeable decrease in % carbon and % of cobalt compared to **(2)**. Chlorine peak in the EDX spectrum of CoCNC@SiO_2_ **(3)** entirely disappeared, indicating the severe decomposition of complex **(1)** at 850 °C. EDX mapping of nitrogen (Figs. S4 and S5) shows 10.53 and 4.12 weight % for **(2)** and **(3)**, respectively, indicating successful doping of nitrogen. In addition, elemental analysis (Table S2) shows 8.316% and 5.36% of nitrogen in **(2)** and **(3)**, respectively.

#### Raman spectroscopy

**Figure 4:**
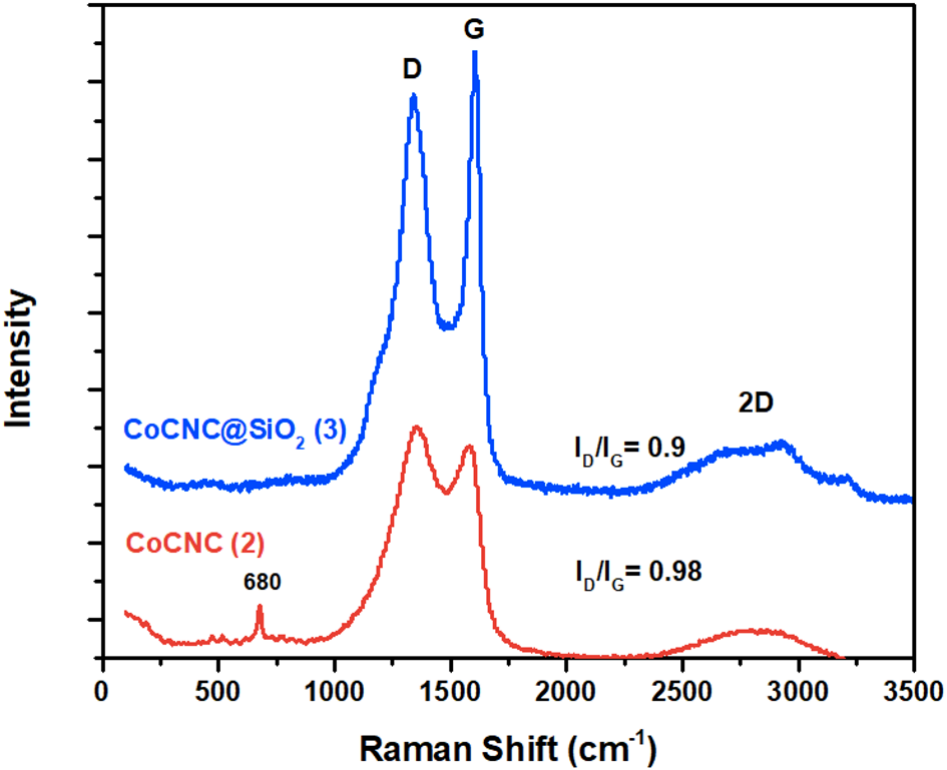
Raman spectra of the pyrolytic products CoCNC **(2)** and CoCNC@SiO_2_ **(3)**.

Raman spectra of **(2)** and **(3)** are shown in Fig. 4. The two characteristic bands named G band and D band are observed in the spectra of carbon nanostructures. The observed D band appears at the range1340–1370 cm^−1^ due to *sp*^3^ disordered or defected graphitic carbon, while G band located at the range 1580–1610 cm^-1^ is due to *sp*^2^ ordered graphitic carbon^38^. Although the position of G band usually appears at 1576–1580 cm^−1^. G band of CoCNC **(2)** and CoCNC@SiO_2_ **(3) **appears at 1590 and 1610 cm^-1^, respectively. The 10–30 cm^−1^ upshift in G band is due to different factors, such as defect, strain, doping and number of layers^39^. D band of CoCNC@SiO_2_ **(3)** appears at ~ 1350 cm^−1^ which is consistent with D band of defect graphite^40^, while CoCNC **(2)** shows D band at 1370 cm^−1^. The shift in D band can be attributed to the presence of oxygen-containing functional groups (as indicated by XPS) which leads to different bond distance of C–C and eventually structural distortion of graphene^41^. The ratio of disordered to graphitic carbon represented by the ratio of the intensities I_D_/I_G_, is a measure of the degree of graphitization^42^. CoCNC@SiO_2_ **(3)** shows slightly higher graphitization degree (lower defection degree) I_D_/I_G_ = 0.9 compared with CoCNC **(2)**. The third characteristic peak of graphite and graphene is the 2D band. Its width and shape depend on the stacking order of the graphene sheets along the c-axis as well as on the number of layers^43^. Both pyrolytic products show a broad 2D band at about 2750 cm^−1^ with relatively low intensities due to the relatively large number of graphene layers^39^. Additional sharp peak at 680 cm^−1^ observed for CoCNC **(2)**, is accompanied by two weaker peaks at ~ 613 cm^−1^ and ~ 516 cm^−1^. This is characteristic of Raman active modes of microcrystalline and nanocrystalline Co_2_O_3_^44^.

#### X-ray photoelectron spectroscopy (XPS)

**Figure 5:**
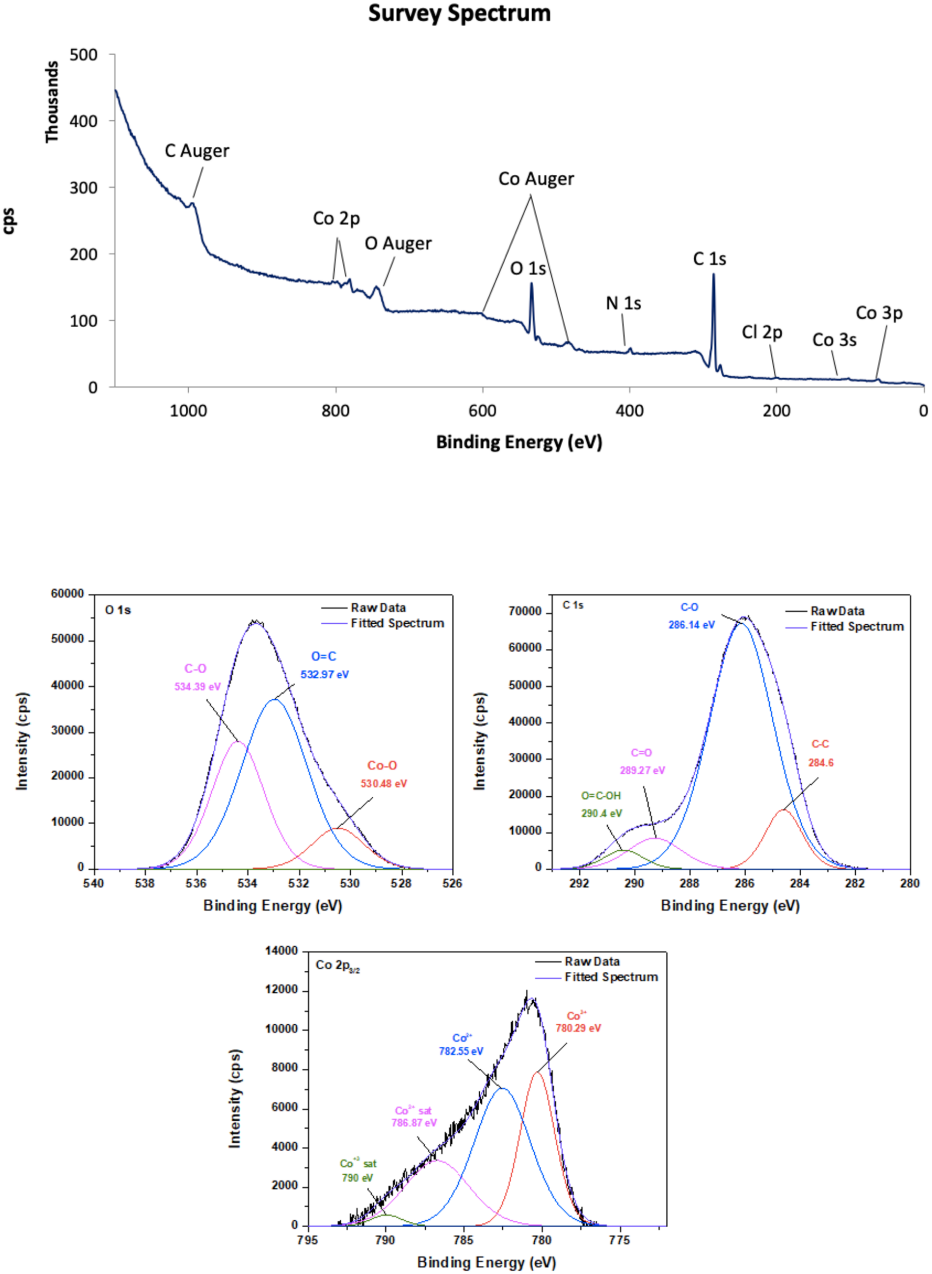
XPS survey of CoCNC **(2)**, C 1*s*, O 1*s* and Co 2*p*_3/2_ spectra.

**Figure 6:**
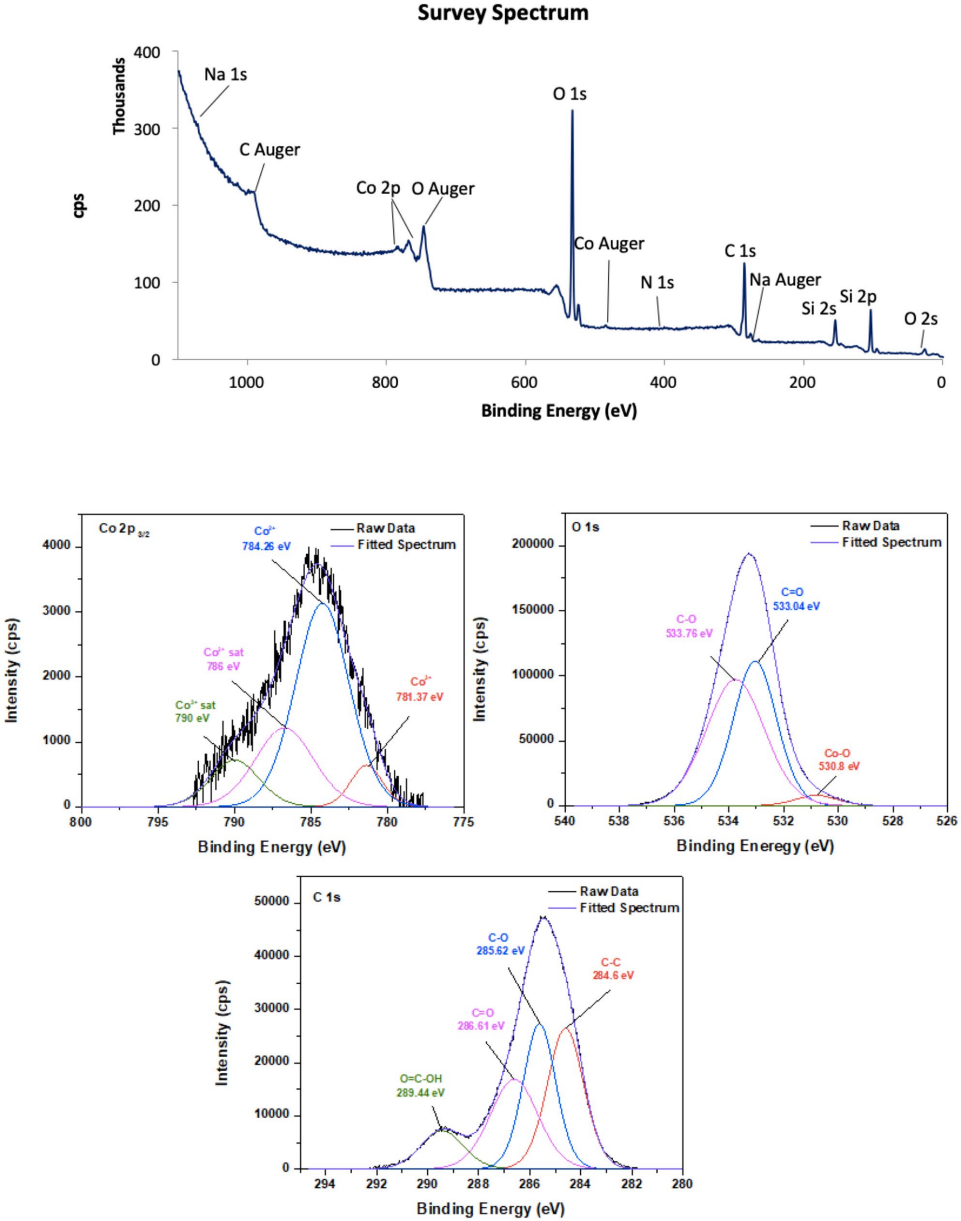
XPS survey of CoCNC@SiO_2_ **(3)**, C 1*s*, O 1*s* and Co 2*p*_3/2_ spectra.

XPS survey and high-resolution spectra of carbon materials are shown in Fig. 5 for CoCNC@SiO_2_ **(2)** and Fig. 6 for CoCNC@SiO_2_**(3)**. XPS survey confirms the existence of carbon, cobalt and oxygen of nanocomposites, and the appearance of chlorine in CoCNC **(2)** which is consistent with EDX results. In addition, detectable nitrogen content is shown, indicating the successful doping of nitrogen. The source of nitrogen can be from bipyridine ligand. The high-resolution of C *1s* of all pyrolytic products is deconvoluted to four individual curves. The curve assigned to *sp*^2^ hybridized carbon appears at 284.6 eV in all carbon materials^37^. However, the amount of C–C/C=C bond on the surface of carbonaceous materials differs due to the variation of pyrolysis conditions. The three signals appeared in C 1*s* spectra around 285–286, 286–289 and 289–290 eV are attributed to C-O /or amorphous carbon, C=O and COOH in the material surfaces, respectively^45,46^. Furthermore, the positions of the peaks obtained from the deconvolution of O 1*s* spectra confirm the presence of different oxygen species. The peak located at 530.48 and 530.8 eV in O 1*s* spectra of CoCNC **(2)** and CoCNC **(3)** respectively, is attributed to the binding energy of Co–O bond^47^, while the peak centered at 533 eV and at 534 eV is attributed to C=O and C–O groups, respectively^46–48^. The deconvoluted Co 2*p*_3/2_ spectra reveal the oxidation states of cobalt species. Although Co^2+^ and Co^3+^ appear at close binding energy, they can shift when Co_3_O_4_ is present^49^. It is noticeable that both carbon materials have a mixture of Co^2+^ and Co^3+^ with different ratios. Co^3+^ peak is observed around 780–781 eV accompanied with a relatively weak satellite peak around 790.0 eV^49^, whereas the peak at 782–784 eV is attributed to Co^2+^^50^. The percentage of C–C in CoCNC **(2)** calculated from the area under the curve is 8.13% which is lower than that of CoCNC@SiO_2_ **(3)** by about 50%, while the percentage of C–O is 57.57%, higher than that of CoCNC@SiO_2_ **(3)** which accounts for only 13%. C=O and O=C–OH groups in CoCNC@SiO_2_ **(3)** are 4.51% and 11.92%, respectively, which are about two-fold higher than that of CoCNC **(2)**. The variation of functional groups percentages on the surface indicates the effect of pyrolysis conditions on surface functionalization of carbon materials.

#### X-ray powder diffraction (XRD)

**Figure 7:**
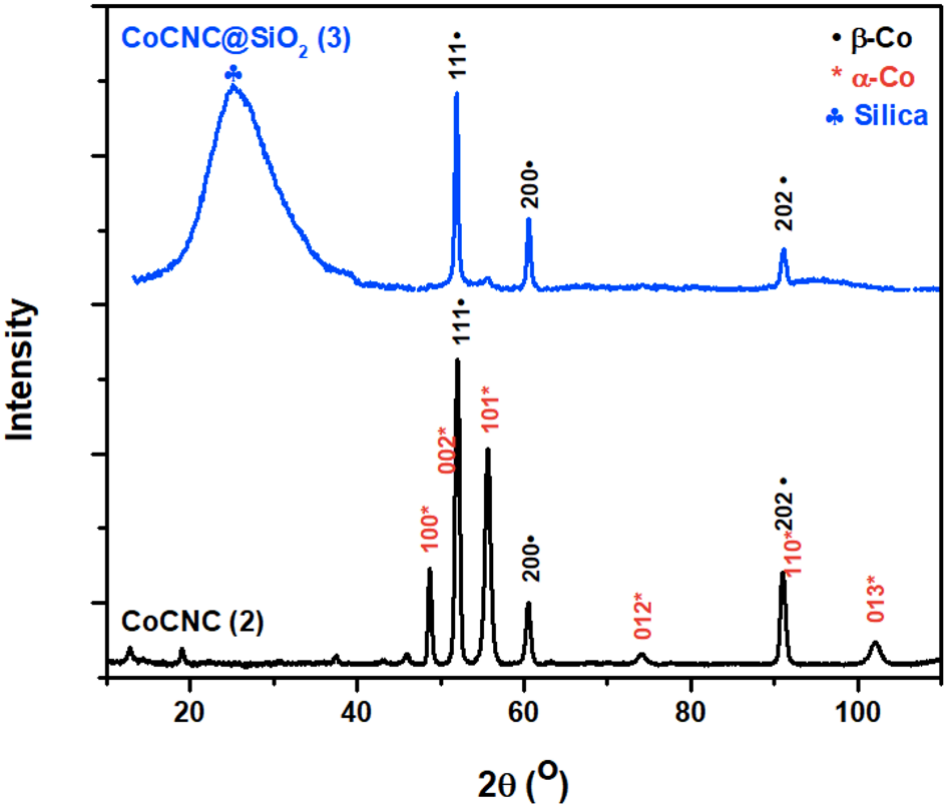
XRD spectrum indicates that CoCNC **(2)** contains hexagonal α-Co and cubic β-Co phases, while CoCNC@SiO_2_ **(3)** has only β-Co phase and a broad silica peak.

**Figure 8:**
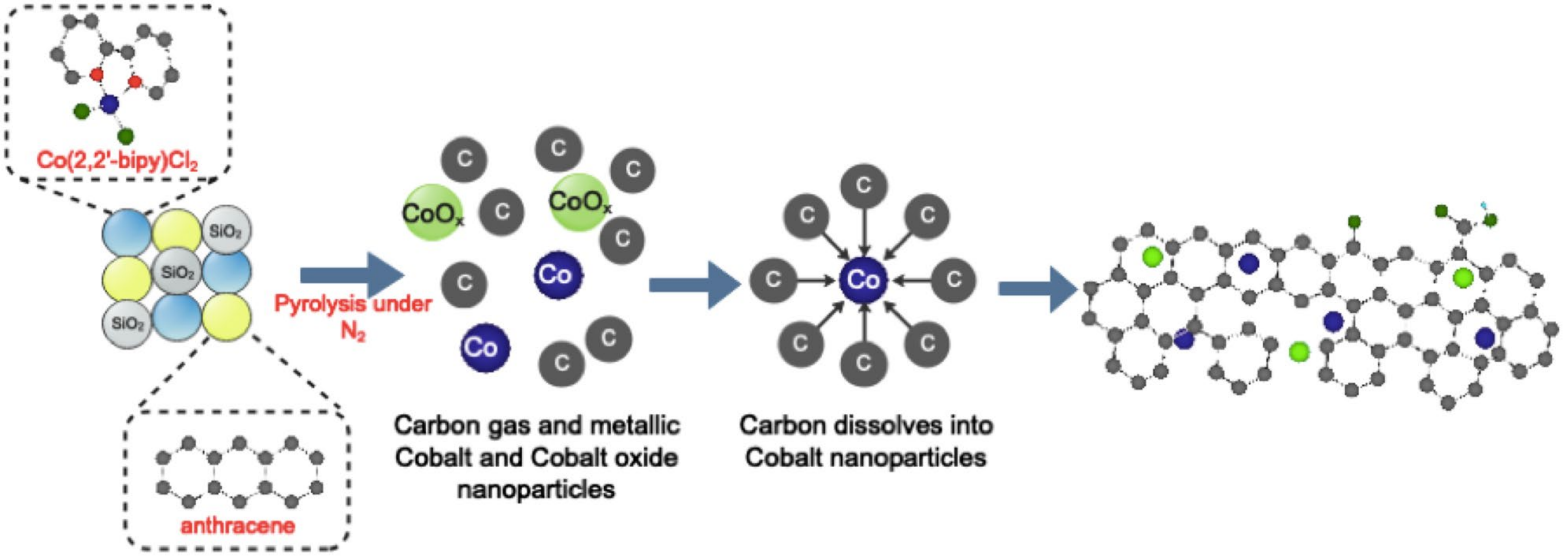
The proposed growing mechanism suggests dissociation of cobalt complex and anthracene into carbon gas and other gaseous products which reduce cobalt (II) to metallic cobalt that acts as a graphitization catalyst.

Figure 7 shows that cobalt existed in a metallic state. This indicates that some cobalt ions are reduced during pyrolysis to zero-state cobalt crystallite and acted as a catalyst for graphitization of carbons obtained from decomposed organic ligands and anthracene. The proposed mechanism is illustrated in Fig. 8. The two phases hexagonal α-Co and cubic β-Co are detected in CoCNC **(2)**, while CoCNC@SiO_2_ **(3)** has only β-Co. A broad peak of **(3)** at 25.55° is attributed to the reflection of silica. This coincides with the fact that β-Co is thermodynamically more stable at high temperature^51^. Based on the weight percentage calculated by Rietveld Refinement, CoCNC **(2)** which is prepared under lower temperature has α-Co as a major phase. The average crystal size of cobalt nanoparticles is calculated using Scherrer formula

1 $$\tau=\frac{0.9 \lambda}{\beta\mathrm{ cos \theta}}$$

where $\tau$ is the average particle size, $\lambda$ is the x-ray wavelength, $\beta$ is the full width at half maximum and $\theta$ is the diffraction angle. The cobalt particle size ranges from 21 to 25 nm and β-Co/α-Co weight ratios are shown in Table 1.

**Table 1:** The crystalline phase, particle size and β-Co:α-Co estimated from XRD analysis.

Sample	Crystalline phase	Particle size	β-Co/α-Co
CoCNC **(2)**	β-Co	21 nm	0.567/1
	α-Co	22 nm	
CoCNC@SiO_2_ **(3)**	β-Co	25 nm	1/0
	SiO_2_	2 nm	

#### FTIR spectra

**Figure 9:**
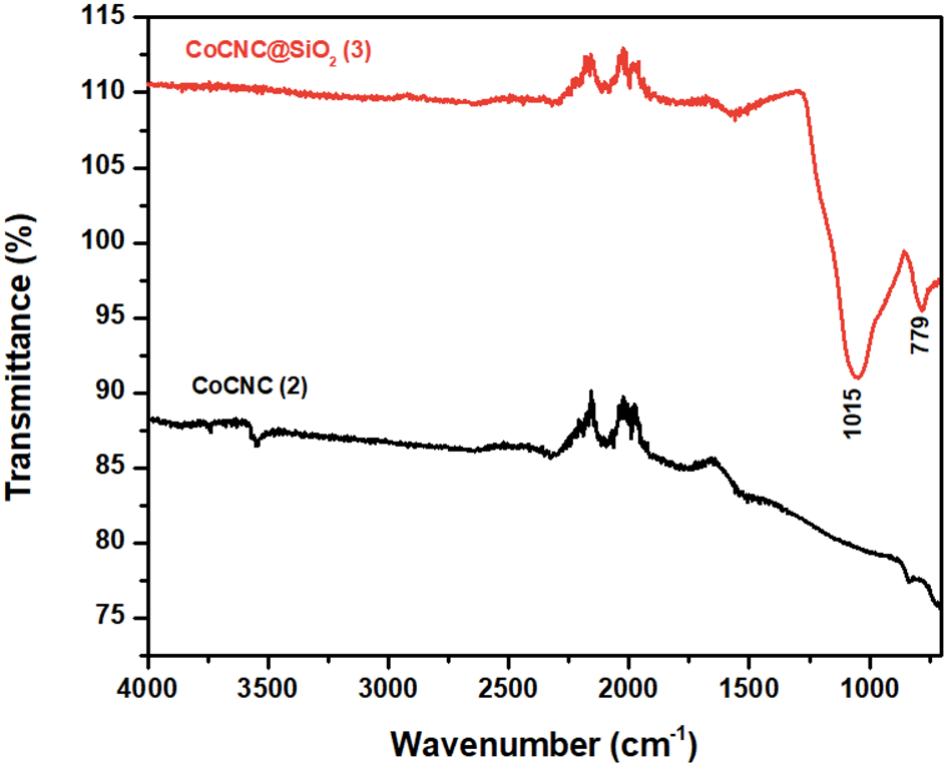
FTIR spectra of CoCNC **(2)** and CoCNC@SiO_2_ **(3)**.

The FTIR technique was used as a probe to investigate the appearance of functional groups in the obtained carbon nanostructures. The two main bands in CoCNC@SiO_2_ **(3)** spectrum are characteristic to Si–O–Si group (Fig. 9). The broad band at 1015 cm^−1^ is assigned to the asymmetric stretching vibration of Si–O and the band at 779 cm^−1^ is due to the bending vibration of Si–O^52^. There is a weak peak at 3550 cm^−1^ attributed to –OH or –NH functional group for CoCNC **(2)** but not present in **(3)**. Also, there is a weak peak at 1550 cm^−1^ for **(3)** and a much weaker one at 1510 cm^−1^ for **(2)** attributable to C–C vibration of aromatic rings.

**Figure 10:**
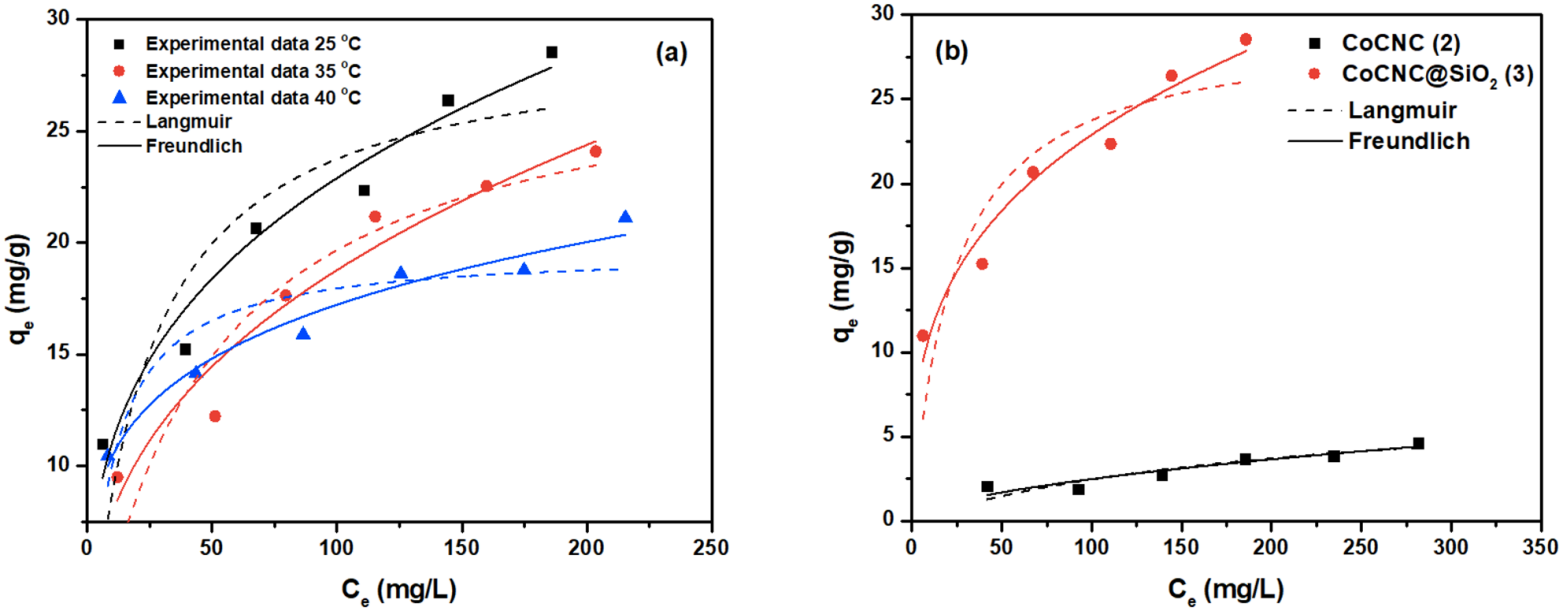
Non-linear fit to Langmuir and Freundlich models for the adsorption of Basic Violet 3 onto CoCNC@SiO_2_ **(3)** (a) and a comparison of the adsorption isotherms of CoCNC **(2)** and CoCNC@SiO_2_ **(3)** at 25 °C.

#### Surface area and porosity

The adsorption/desorption isotherms of the nanocarbon samples **(2)** and **(3)** were fitted to the Brunauer-Emmett Teller (BET) model, and the obtained isotherms of the both nanocarbons were showed lower and moderate BET surface area of 3.4 and 97.8 m^2^ g^−1^ respectively as shown in Fig. S6. The pore size distribution of the nanocarbons **(2)** and **(3)** were calculated with a BJH fitting method and nanocarbon sample **(2)** showed poor nitrogen gas uptake, which clearly indicate nonporous nature of the nanocarbon sample. Instead, nanocarbon sample **(3)** showed a moderate nitrogen uptake with an unusual H1-type hysteresis loop characteristics according to the IUPAC classifications^53^. The BJH pore-size distribution plot showed a broad mesopore-size distribution with an average pore width of 53.5 Å with the pore volume ranging between 0.024–0.027 cm^3^ g^−1^ (Fig. S7).

### Water treatment

CoCNC **(2)** and CoCNC@SiO_2_ **(3)** are tested as adsorbents of Basic Violet 3 dye. As shown in Fig. 10, **(2)** has a poor adsorption behavior at 25 °C. It can be attributed to the low surface area of **(2)**, while **(3)** has much larger surface area and good adsorption capacity^54,55^.

#### Effect of initial concentration

Figure S8 illustrates the effect of dye initial concentration on the adsorption capacity of CoCNC@SiO_2_ **(3)**. The amount of dye adsorbed increases as the initial dye concentration increases, due to the fact that dye molecules adsorbed on the outer surface independently at low concentration, while at high concentration, dye molecules diffuse into the inner pores, causing an increase in capacity^56^. In contrast, the % removal of dye is very high at low concentration because of the availability of vacant adsorption sites for binding Basic Violet 3 ion. As initial concentration increases, it dropped dramatically then decreased gradually due to the gradual occupation of binding sites^57^.

#### Effect of adsorbent dosage

The dosage effect of adsorbent **(3)** on the adsorption capacity was shown in Fig. S9. The quantity of dye adsorbed increased significantly when the dosage is increased from 3 to 4 g L^−1^ due to the increased number of adsorption sites with increasing dosage^58^. Then it remains almost unchanged when it is increased to 5 g L^−1^. It can be attributed to the fact that aggregation decreases the exposed surface area and lengthens the diffusion path of dye^59^.

#### Effect of pH

pH of aqueous solution plays an important role during the adsorption of ionic dyes because pH influences the ionization of the active groups of both adsorbent and adsorbate. The adsorption of Basic Violet 3 on **(3)** was studied in the pH range from 3 to 10 (Fig. S10). The adsorption capacity increased from 9 mg g^−1^ at pH 5 to16 mg g^−1^ at pH 9. In an acidic medium (pH < 7), the adsorption capacity is lower than that at pH ≥ 7 due to the competition of H^+^ ions with cationic Basic Violet 3. Nevertheless, even at acidic medium, the Basic Violet 3 still adsorbed on CoCNC@SiO_2_ **(3)**, suggesting that the electrostatic interaction is not the only mechanism involved in the adsorption process^60^. Therefore, π–π interactions between **(3)** and Basic Violet 3 plays a crucial role in the adsorption process.

#### Adsorption isotherm

The experimental Basic Violet 3 adsorption data by **(2)** and **(3)** obtained at different temperatures were plotted using linear and non-linear Langmuir and Freundlich isotherm models (Fig. 10). For Langmuir Isotherm^61^, kinetic consideration was taken when this empirical model is constructed. It is based on the assumption that adsorption sites are equivalent and identical and only one layer of adsorbed molecules is formed (monolayer adsorption), with no interaction between the adsorbed molecules^8^.

Where ${\mathrm{q}}_{\mathrm{e}}$ (mg g^−1^) is the amount of adsorbate adsorbed at equilibrium, ${\mathrm{q}}_{\mathrm{max}}$ (mg g^−1^) is the maximum adsorption capacity, ${\mathrm{C}}_{\mathrm{e}}$ (mg L^−1^) is the concentration of adsorbate at equilibrium and ${\mathrm{K}}_{\mathrm{L}}$ (L mg^−1^) is the Langmuir adsorption constant^61^. The dimensionless separation factor of Langmuir isotherm model ${\mathrm{R}}_{\mathrm{L}}$ is determined by Webber and Chakkravorti, where R_L_ is 1/K_L_C_o_^8,62^. Where ${\mathrm{C}}_{\mathrm{o}}$ (mg L^−1^) is the initial concentration of the adsorbate. Separation factor determines if the adsorption is favorable (0 < ${\mathrm{R}}_{\mathrm{L}}$ < 1), unfavorable (${\mathrm{R}}_{\mathrm{L}}$ > 1). Irreversible (${\mathrm{R}}_{\mathrm{L}}=0)$ or linear (${\mathrm{R}}_{\mathrm{L}}$ = 1)^63^.

Freundlich isotherm^64^ is an empirical adsorption model formulated to describe the non-ideality and reversibility of the adsorption process. This model is employed to describe multilayer adsorption, not limited to the formation of a monolayer. It suggested unequal affinities and energy distribution over the heterogeneous adsorption sites^65^. At present, Freundlich isotherm is widely employed in heterogeneous systems. The value of 1/n can be used to characterize surface heterogeneity^8^.

Freundlich constant K_f_ ((mg g^−1^)(L mg^−1^)1/n) indicates the adsorption capacity and n indicates the adsorption intensity of the adsorbent. Freundlich constants can be extracted from the plot of lnq_e_ against lnC_e_. The intercept value is lnK_f_, where the slop is the value of 1/n. The value of the latter ranges from 0 to 1. When the value of 1/n is closer to zero, it means that the binding surface is more heterogeneous. In general, the strength of adsorption intensity can be indicated from the value of n. If the value is higher than unity, the adsorbate is favorably adsorbed on the adsorbent’s surface. The higher the value of n the stronger the adsorption intensity^8,64^.

Correlation coefficients R^2^, Chi^2^, and SSR values, were used to find the best-fitted isotherm to the experimental data. Moreover, isotherm parameters were obtained from the isotherm models shown in Fig. 10, Fig. S11 and Fig. S11. As statistical parameters show (Table 2), the experimental data has a good agreement with the Freundlich model. CoCNC@SiO_2_ **(3)** has higher capacity and thus is better adsorbent of Basic Violet 3 than CoCNC **(2)**. This was also expected from BET surface area measurements. Thus, the silica particles support that we have used in preparing powder **(3)** has played a major role in improving capacity compared to micro sheets **(2)**.

**Table 2:** Langmuir and Freundlich isotherm parameters for the adsorption of Basic Violet 3 dye onto CoCNC **(2)** and CoCNC@SiO_2_ **(3)** at 25 °C.

Langmuir			Freundlich		
Parameter	Linear $\frac{{\mathrm{C}}_{\mathrm{e}}}{{\mathrm{q}}_{\mathrm{e}}}=\frac{{\mathrm{C}}_{\mathrm{e}}}{{\mathrm{q}}_{\mathrm{max}}}+\frac{1}{{\mathrm{q}}_{\mathrm{max}}{\mathrm{K}}_{\mathrm{L}}}$	Non-linear ${\mathrm{q}}_{\mathrm{e}}=\frac{{\mathrm{q}}_{\mathrm{max}}{\mathrm{K}}_{\mathrm{L}}{\mathrm{C}}_{\mathrm{e}}}{1+{\mathrm{K}}_{\mathrm{L}}{\mathrm{C}}_{\mathrm{e}}}$	Parameter	Linear ${\mathrm{lnq}}_{\mathrm{e}}={\mathrm{lnK}}_{\mathrm{f}}+\frac{1}{\mathrm{n}}{\mathrm{lnC}}_{\mathrm{e}}$	Non-linear ${\mathrm{q}}_{\mathrm{e}}={\mathrm{K}}_{\mathrm{f}}({{\mathrm{C}}_{\mathrm{e}})}^{\frac{1}{\mathrm{n}}}$
**CoCNC (2)**					
q_max_ (mg g^−1^)	6.908	7.553	K_F_ (mg g^−1^)(L mg^−1^)^1/n^	0.3165	0.19
K_L_ (L mg^−1^)	0.0057	0.0048	n	0.455	1.796
R^2^	0.677	0.776	R^2^	0.741	0.852
Chi^2^	0.8615	0.260	Chi^2^	0.8905	0.171
SSR	290.5252	1.0411	SSR	0.1337	0.686
R_L_	0.3672	0.694			
**CoCNC@SiO**_**2**_** (3)**					
q_max_ (mg g^−1^)	21.958	19.654	K_F_ (mg g^−1^)(L mg^−1^)^1/n^	6.604	6.316
K_L_ (L mg^−1^)	0.0473	0.105	n	4.812	4.591
R^2^	0.97	0.754	R^2^	0.97	0.962
Chi^2^	0.991	3.621	Chi^2^	0.988	0.551
SSR	1.098	2.224	SSR	0.007	2.205
R_L_	0.065	0.0307			

#### Adsorption kinetics

The investigation of adsorption kinetics of water pollutants is needed to explain the adsorption mechanism and to design suitable water treatment plants^66^. To estimate the time required to attain an equilibrium, adsorption capacity is plotted against time at 25 °C (Fig. S13). Adsorption increases sharply in the first 120 min. The high adsorption rate at initial contact time is attributed to the availability of binding sites on the adsorbent’s surface^59^. Then, the rate decreases and the capacity remain constant at equilibrium after 24 h. Two kinetic models: pseudo-first-order kinetic and pseudo-second-order kinetic models are applied to describe the adsorption process of CoCNC@SiO_2_ **(3)**.

The empirical pseudo-first-order kinetics (Lagergren) is widely used to describe the rate of the adsorption process in solid–liquid systems. It assumed that one adsorption site is occupied with one molecule^67^. The rate constant k_1_ and capacity q_e_ can be obtained from the slope and intercept of the linear plot of log(q_e_ − q_t_) against t; where q_e_ and q_t_ (mg g^−1^) are the amount of adsorbate adsorbed at equilibrium and at time t^68^. While pseudo-second-order kinetic assumes that one adsorbing molecule reacts with two adsorption sites^67^. The values of q_e_ and rate constant k_2_ can be found from the slope of the plot of t/q_t_ versus t; as the slope is the value of 1/q_e_ and the intercept is 1/k_2_(q_e_)^2^.

**Figure 11:**
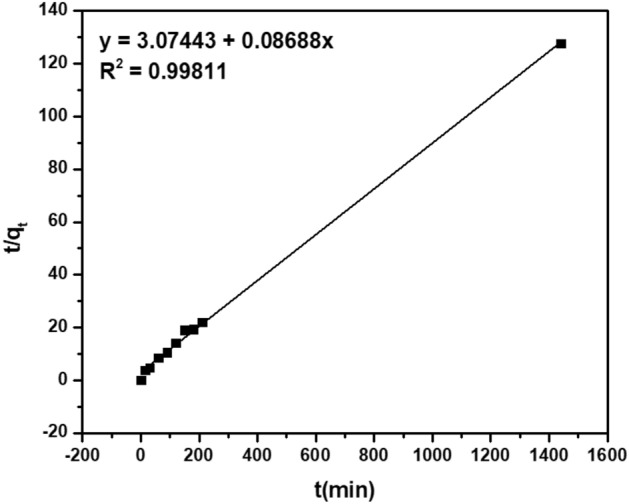
Pseudo-second-order linear plot for the adsorption of Basic Violet 3 onto CoCNC@SiO_2_ **(3)** at 298 K.

Comparing the correlation coefficient values R^2^ of linear and non-linear approaches (Figs. 11, S14), it was found that R^2^ for the pseudo-second-order model is 0.99 and 0.94 respectively. They are higher than that for pseudo-first-order model which is 0.84 and 0.87. As a result, the adsorption of Basic Violet 3 dye onto CoCNC@SiO_2_ (**3**) is found to fit well the pseudo-second-order model^68^.

This means that the rate of adsorption capacity increases with increase in concentration of the adsorbate and the amount of the adsorbent in the mixture. In addition, the pseudo-second-order rate constant obtained by linear and non-linear approaches is 0.0024 and 0.0026 g mg^−1^ min^−1^, respectively.

#### Thermodynamic parameters

The value of change of entropy $\Delta \mathrm{S}$ and the change of enthalpy $\Delta \mathrm{H}$ can be determined from the following linear equation, Eq. (2):

2 $${\mathrm{lnK}}_{\mathrm{e}} = \frac{\Delta \mathrm{S}}{R}-\frac{\Delta \mathrm{H}}{RT}$$

where, R is the ideal gas constant, and T (K) is the absolute temperature and K_e_ is the partition (or distribution) coefficient defined as the ratio of the concentration of adsorbate on the adsorbent C_r_ (mg L^−1^) to its concentration in the solution C_e_ (mg L^−1^) at equilibrium.

From the plot of lnK_e_ versus 1/T (Fig. S15), the values of ΔH and ΔS can be obtained from the slope and intercept, respectively. Therefore, the value of the change of Gibb’s free energy ΔG at a given temperature can be calculated from ΔH − TΔS. The ∆H value is − 26.258 kJ mol^−1^ which indicates the exothermic nature of the adsorption process, which agrees with isotherm results where q_max_ decreased with temperature. It may be attributed to the assumption that high temperatures may weaken the adsorption bonds created between dye molecules and binding sites^69^. In addition, the ∆S value is − 86.374 J mol^−1^ K^−1^ which reflects the nonspontaneous nature of the adsorption process within the range of temperature studied, whereas the increase in the value of ΔG as temperature increases (− 0.519 kJ mol^−1^ at 298 K, 0.344 at 308 K and 0.776 kJ mol^−1^ at 313 K) indicates that the adsorption process is more favorable at lower temperatures.

#### Column study

**Figure 12:**
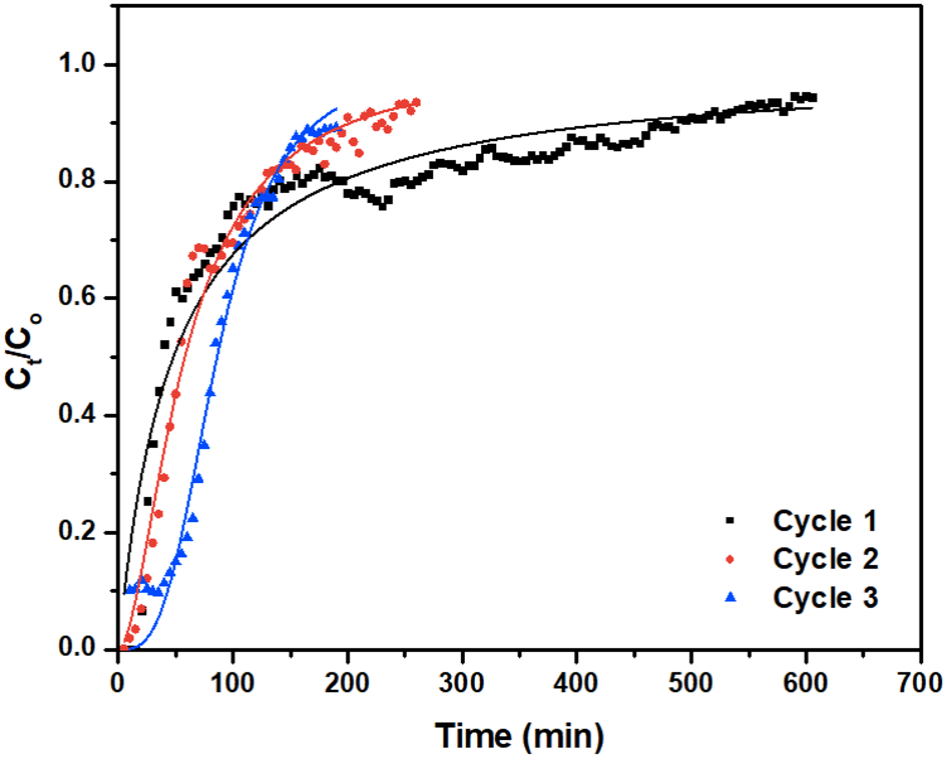
Nonlinear fit Yan et al. column model for adsorption of Basic Violet 3 onto CoCNC@SiO_2_ **(3)**.

Investigation of regeneration capacity was conducted using a fixed-bed adsorption column in a continuous adsorption system using HCl as a cost-effective and proton-exchange eluting agent for desorbing cationic dyes from adsorbents. The breakthrough curves for the 3 cycles of Basic Violet 3 adsorption in the fixed-bed column is shown in Fig. 12.

The breakthrough curves for the first and second cycles show that the curves break immediately at the given flow rate, while the breakthrough point for the third cycle appears after a short delay. For the desorption process, the first desorption cycle requires about 170 mL of 0.1 M HCl solution, with a flow rate of 1 mL min^−1^ to fully desorb dye cations. In addition, the relatively high concentrate eluate (50 mg L^−1^) can be further processed easily for Basic Violet 3 recovery and reusability.

Adsorption capacity calculated using breakthrough curve method^70–72^ for the first cycle is 12.55 mg g^−1^, then it drops to 8.30 mg g^−1^ for the second and third cycles. After regeneration of the first cycle, HCl displaced the cationic Basic Violet 3 dye but also dissolved some of thin carbon coated cobalt nanoparticles. The capacity drops in going from first to second cycle. This indicates that cobalt nanoparticles contribute to adsorption of Basic Violet 3 cations. However, regeneration after the second cycle is efficient in displacing only the dye as the capacity in the third cycle remains the same as that of second cycle.

#### Adsorption mechanism

**Figure 13:**
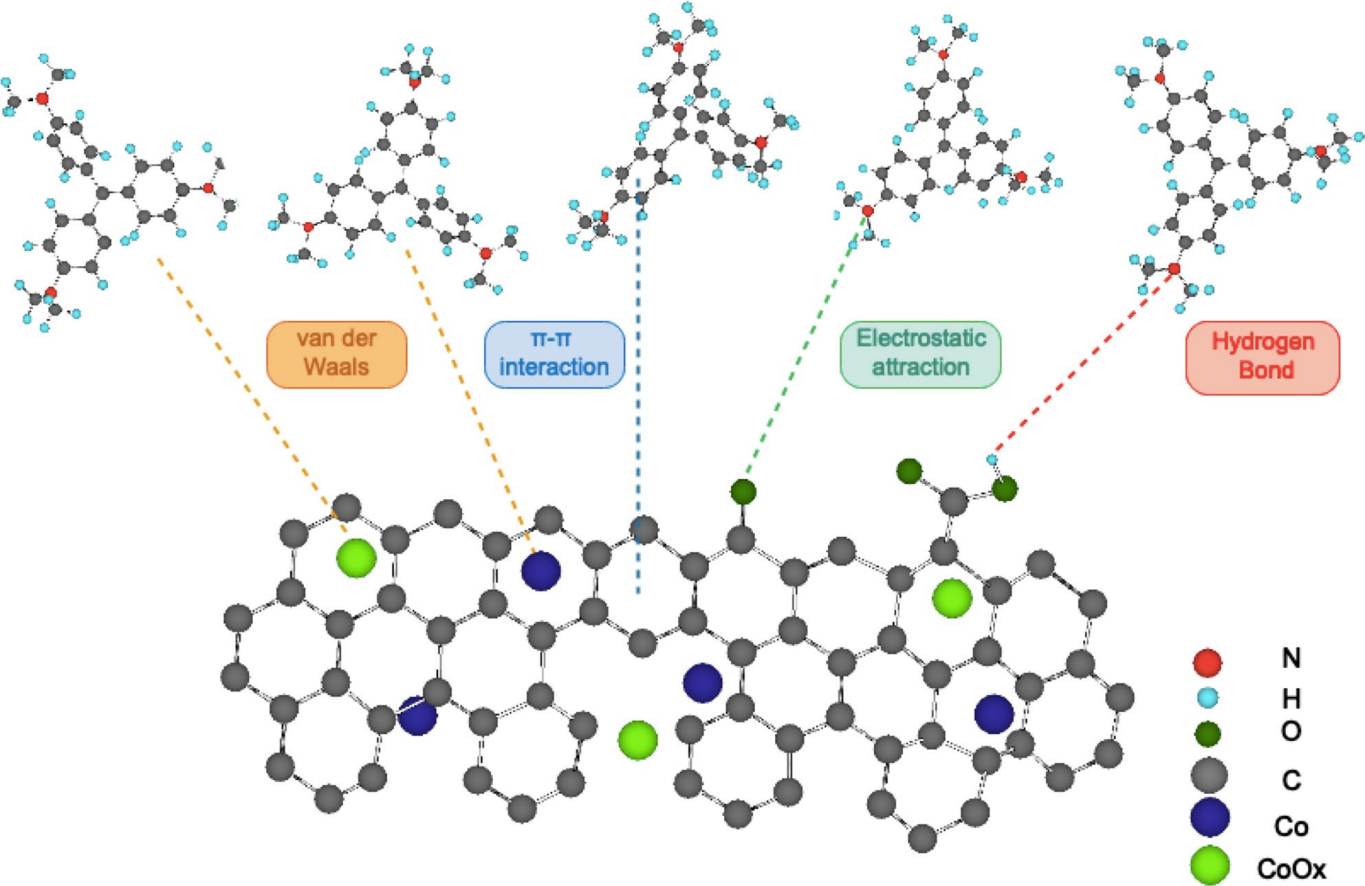
Proposed mechanism of Basic Violet 3 adsorption on CoCNC **(2)** and CoCNC@SiO_2_ **(3)**, showing the possible hydrogen bonds between hydroxyl and carboxyl groups of carbon nanocomposite and nitrogen atoms of Basic Violet 3, π-π interaction between the aromatic rings of graphitized carbon and the dye, and the possible van der Waals bond between graphitic metallic cobalt and cobalt oxide nanoparticles.

FTIR of **(3)** was carried out before and after adsorption of Basic Violet 3 (Fig. S16). The two bands assigned to Si–O–Si at 1015 cm^−1^ and 779 cm^−1^ remain intact after adsorption, suggesting that Si–O–Si group of **(3)** is not involved in chemical interaction with adsorbate. Two new peaks appeared at 1589 cm^−1^ and 1366 cm^−1^ after adsorption of dye. They are assigned to stretching vibration C=C of aromatic ring and C-N of tertiary amine of Basic Violet 3 respectively, supporting the uptake of the dye^73^. Except for the two bands assigned to Basic Violet 3, there is no new peak appeared after adsorption. This suggests that the adsorption process occurs via physical bonds such as π-π stacking, hydrogen bond, electrostatic attraction and van der Waals force between aromatic rings of Basic Violet 3 and carbon nanostructures (Fig. 13). Moreover, to investigate the mechanism of adsorption, adsorbents were treated with hydrochloric acid to remove cobalt species. The adsorption capacity for CoCNC@SiO_2_ **(3)** dropped from 18.96 to 7.51 mg g^−1^, suggesting the involvement of cobalt nanoparticles in the adsorption process.

#### Column kinetic study and reusability

Description of the column behavior and parameters essential for designing and optimizing a continuous adsorption system are provided by applying three different column models, Thomas, Yoon-Nelson, and Yan et al. models^74,75^.

Thomas model (Table 3) considers Langmuir kinetics of adsorption–desorption, negligible axial dispersion in the column and follows pseudo- second-order reversible kinetics.

**Table 3:** Column kinetic parameters for the adsorption of Basic Violet 3 onto CoCNC@SiO_2_ **(3)** for the three cycles.

Thomas		Yoon-Nelson		Yan et al.	
$\frac{{\mathrm{C}}_{\mathrm{e}}}{{\mathrm{C}}_{\mathrm{o}}}\text{=}\frac{1}{1+{\mathrm{e}}^{\frac{{\mathrm{k}}_{\mathrm{T }}}{\mathrm{Q}}({\mathrm{q}}_{\mathrm{T}}\mathrm{ m}.{\mathrm{C}}_{\mathrm{o }}\mathrm{V})}}$		$\frac{{\mathrm{C}}_{\mathrm{e}}}{{\mathrm{C}}_{\mathrm{o}}}\text{=}\frac{1}{1+{\mathrm{e}}^{{\mathrm{k}}_{\mathrm{YN}} (\mathrm{T}-\mathrm{t})}}$		$\frac{{\mathrm{C}}_{\mathrm{e}}}{{\mathrm{C}}_{\mathrm{o}}}\text{=1-}\frac{1}{1+{(\frac{{\mathrm{Q}}^{2}\mathrm{t}}{{\mathrm{k}}_{\mathrm{Y}}{\mathrm{q}}_{\mathrm{Y}}\mathrm{m}})}^{\frac{{\mathrm{k}}_{\mathrm{Y}}{\mathrm{C}}_{\mathrm{o}}}{\mathrm{Q}}}}$	
**1st cycle**					
k_T_ (mL min^−1^ mg^−1^)	0.131	k_YN_ (min^−1^)	0.006	k_Y_ (mL min^−1^ mg^−1^)	0.019
q_T_ (mg g^−1^)	3.394	q_YN_ (mg g^−1^)	3.400	q_Y_ (mg g^−1^)	4.820
		$\mathrm{T}$(min)	34.043		
R^2^	0.641	R^2^	0.641	R^2^	0.916
Chi^2^	0.011	Chi^2^	0.011	Chi^2^	0.002
SSR	1.406	SSR	1.406	SSR	0.328
**2nd cycle**					
k_T_ (mL min^−1^ mg^−1^)	0.489	k_YN_ (min^-1^)	0.024	k_Y_ (mL min^−1^ mg^−1^)	0.035
q_T_ (mg g^−1^)	6.833	q_YN_ (mg g^−1^)	6.83	q_Y_ (mg g^−1^)	3.294
		$\mathrm{T}$(min)	68.341		
R^2^	0.884	R^2^	0.884	R^2^	0.978
Chi^2^	0.008	Chi^2^	0.008	Chi^2^	0.001
SSR	0.435	SSR	0.435	SSR	0.082
**3rd cycle**					
k_T_ (mL min^−1^ mg^−1^)	0.686	k_YN_ (min^-1^)	0.034	k_Y_ (mL min^−1^ mg^−1^)	0.063
q_T_ (mg g^−1^)	9.036	q_YN_ (mg g^−1^)	9.036	q_Y_ (mg g^−1^)	2.722
		$\mathrm{T}$(min)	90.364		
R^2^	0.974	R^2^	0.974	R^2^	0.980
Chi^2^	0.002	Chi^2^	0.002	Chi^2^	0.002
SSR	0.088	SSR	0.088	SSR	0.07

Where ${\mathrm{C}}_{\mathrm{o}}$ is the initial concentration of the dye solution while k_T_ (mL mg^−1^ min^−1^) and q_T_ (mg g^−1^) are Thomas rate constant and column capacity respectively. m(g) is the amount of adsorbent in the column and Q (mL min^−1^) is the volumetric flow rate. The constants k_T_ and q_T_  can be obtained from the plot of $\mathrm{ln}[(\frac{{\mathrm{C}}_{\mathrm{o}}}{{\mathrm{C}}_{\mathrm{e}}}\text{)-1]}$ against time t.

Yoon-Nelson model is a relatively simple model (Table 3). k_YN_ (min^−1^) and τ (min) are the rate constant and the necessary time for 50% adsorbate breakthrough, respectively. They can be obtained from the linear plot of ln(C_e_/(C_o_ − C_e_)) against time t.

Since the quantity of adsorbate being adsorbed on the bed is half of the total amount of adsorbate entering the bed within 2 τ periods, the following equation is formulated:

3 $${\mathrm{q}}_{\mathrm{YN}}\text{=}\frac{{\mathrm{q}}_{\mathrm{total}}}{\mathrm{m}}=\frac{\frac{1}{2}{\mathrm{C}}_{\mathrm{o}}(\frac{\mathrm{Q}}{1000})(2\mathrm{T})}{\mathrm{m}}=\frac{{\mathrm{C}}_{\mathrm{o}}\mathrm{QT}}{1000\mathrm{ m}}$$

where ${\mathrm{q}}_{\mathrm{YN}}$ is Yoon-Nelson column capacity, Q (mL min^−1^) is the volumetric flow rate, C_o_ (mg L^−1^) is the initial concentration of the feed solution, m (g) is the total dry mass of adsorbent in the column.

Empirical Yan et al. model considers to overcome the deficiency in Thomas model in predicting the effluent concentration at time zero. Ky (L min^−1^ mg^−1^) and qy (mg g^−1^) are the kinetic rate constant and the maximum adsorption capacity of adsorbent.

Comparing the values of Chi^2^, SSR, and R^2^, we find that Yan et al. model is fitted well with the experimental data. It can best describe the behavior of Basic Violet 3 adsorption in a fixed-bed column packed with **(3)**.

### Comparison of adsorption capacity of (3) with other adsorbents

The maximum adsorption capacities of various adsorbents were compared with that of CoCNC@SiO_2_ **(3)** in Table 4. The comparison indicates that **(3)** has higher adsorption capacity than other adsorbents, such as modified graphene oxide and titanate nanotubes. It is less than the more expensive MWCNT. However, **(3)** has the advantage of being prepared in a simple and cost-effective method. The substrate silica and anthracene were used as 2/3 of the starting materials.

**Table 4:** Comparison of adsorption capacity of **(3)** with other adsorbents.

Adsorbent	Capacity (mg g^−1^)	Reference
Grafted sodium alginate/ZnO/graphene oxide	13.85	^76^
Titanate nanotube	8.36	^77^
Graphene oxide/activated carbon	70	^10^
MWCNTs-OH	988	^78^
Cobalt-carbon@silica nanocomposite	19.6	This study

## Conclusion

Cobalt-carbon nanocomposites, CoCNC **(2)** and CoCNC@SiO_2_ **(3)**, are synthesized by pyrolysis of a mixture of Co(2,2′-bipy)Cl_2_ and anthracene. Based on SEM and TEM images, it was found that pyrolysis under 600 °C forms CoCNC **(2)** with 3D hierarchical carbon architecture decorated with cobalt nanoparticles. Increasing the temperature to 850 °C in presence of silica results in the formation of CoCNC@SiO_2_ **(3)** with graphitized structure around cobalt nanoparticles embedded in the silica matrix. Furthermore, carbon nanostructure is functionalized with different oxygen-containing functional groups, including carboxylic acid group as XPS technique reveals. PXRD spectrum indicates that CoCNC **(2)** contains hexagonal α-Co and cubic β-Co phases, while CoCNC@SiO_2_ **(3)** has only β-Co phase with broad silica peak. The efficiency of **(2)** and **(3)** for removal of Basic Violet 3 from aqueous solution was studied. **(3)** was found better adsorbent than **(2)**. The calculated Langmuir adsorption capacity is about 19.6 mg g^−1^ at 25 °C. Freundlich isotherm and pseudo-second-order kinetic model describe best the adsorption process. Thermodynamic study shows that the adsorption process is exothermic, more ordered process and more favorable at lower temperature as indicated by ∆H, ∆S and ∆G values. Furthermore, column adsorption capacity is 12.55 mg g^−1^ and Yan et al. model adequately described the continuous adsorption process. Three cycles were undertaken for column adsorption, with no noticeable drop in capacity between second and third cycles.

## Supplementary information

Supplementary Information.
