# Supplementary material for: Novel cobalt–carbon@silica adsorbent
Source: Sci Rep. 2020 Oct 29;10:18652. doi: 10.1038/s41598-020-75367-0 (PMC7596546; doi:10.1038/s41598-020-75367-0)
Supplement: Supplementary file 1 — Supplementary Information. [file 41598_2020_75367_MOESM1_ESM.docx]

**Supplementary Information**

**Novel Cobalt-Carbon@Silica Adsorbent**

**Nusaybah Alotaibi^1^, Hassan H. Hammud^1*^, Nasreen Al Otaibi^1^, Syed Ghazanfar Hussain^2^, Thirumurugan Prakasam^3^**

***^1^Department of Chemistry, College of Science, King Faisal University, P.O Box 400 Al-Ahsa 31982, Saudi Arabia.***

***^2^Department of Physics, College of Science, King Faisal University, P.O Box 400 Al-Ahsa 31982, Saudi Arabia.***

***^3^Chemistry Program, New York University Abu Dhabi (NYUAD), Abu Dhabi United Arab Emirates.***

**** E-mail:*** [***hhammoud@kfu.edu.sa***](mailto:hhammoud@kfu.edu.sa)

***Tel: 00966 (0)13 589 9579***

**1.** **Characterization of Co(2,2’-bipy)Cl_2_ (1) by FTIR and Thermal Analysis**

- FTIR spectra of complexes are shown in **Figure S1**. The assignments of the significant IR spectral bands of 2,2-bipyridine and its cobalt complex **(1)** are presented in **Table S1.** (>C=N) and (>C=C<) bands of 2,2’-bipy appear at 1577 cm^-1^ and 1556 cm^-1^. Upon complexation, they shifted to 1598 cm^-1^ and 1560 cm^-1^, indicating the involvement of nitrogen in coordination to Co(II). In addition, absorption peaks assigned to aromatic C-H vibrations are observed in the complex at 3054 cm^-1^ which is identical to that observed in 2,2’-bipyridine, suggesting the successful coordination of 2,2’-bipyridine.


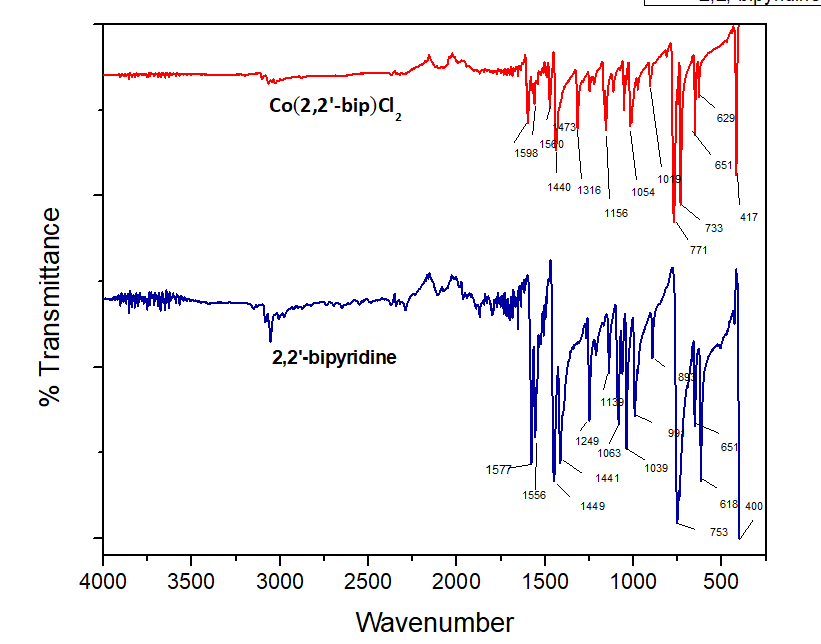


**
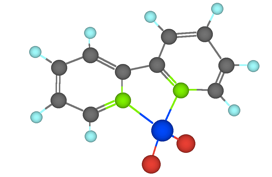
**


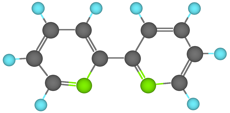


**Figure S1.** FTIR spectrum of Co(2,2’-bipy)Cl_2_ **(1)** and the ligand 2,2’-bipyridine.

**Table S1.** FTIR assignment for complex Co(2,2’-bipy)Cl_2_ and 2,2’-bipy ligand.

| **2,2'-bipy**^1^ | **Co(2,2’-bipy)Cl_2_** | **Assignment** |
| --- | --- | --- |
| 3054 w | 3054 w | aromatic (=C-H) stretching vibration |
| 1577 s  1556 s | 1598 w  1560 w | aromatic (C=C,C=N) ring stretching |
| 1449 s  1414 s  1063 m | 1473 w  1440 m  1054 w | ring stretching + C-H in-plane bending |
| 991 m | 1019 m | C-H out-of-plane bending ring breathing |
| 893 m  753 s | 906 w  771 s | C-H out of plane bending |
| 651 m  618 s | 651 m  629 w | ring bending |

- TGA data (**Figure S2.**) showed that complex (1) is thermally stable up to 279 ^o^C. Above this temperature, the thermogram shows a weight loss of 50.6% (Calc. 51%) between 279 ^o^C and 550 ^o^C which reasonably accounts for the loss of 2,2’-bipyridine ligand. The second stage centred at the temperature of 588 ^o^C shows a weight loss of 11.67% (Calc. 12.39%) which can be attributed to the loss of one chloride ion. Assuming complexation of only one chloride since the energy required to break coordination bond is relatively high.

Based on TGA curve degradation pattern which starts at 300 ^0^C. We have chosen an intermediate stage to be at 300 for 2h in the pyrolysis experiment of (1) to give (**2**) at 600 ^0^C and (**3**) at 850 ^0^C.


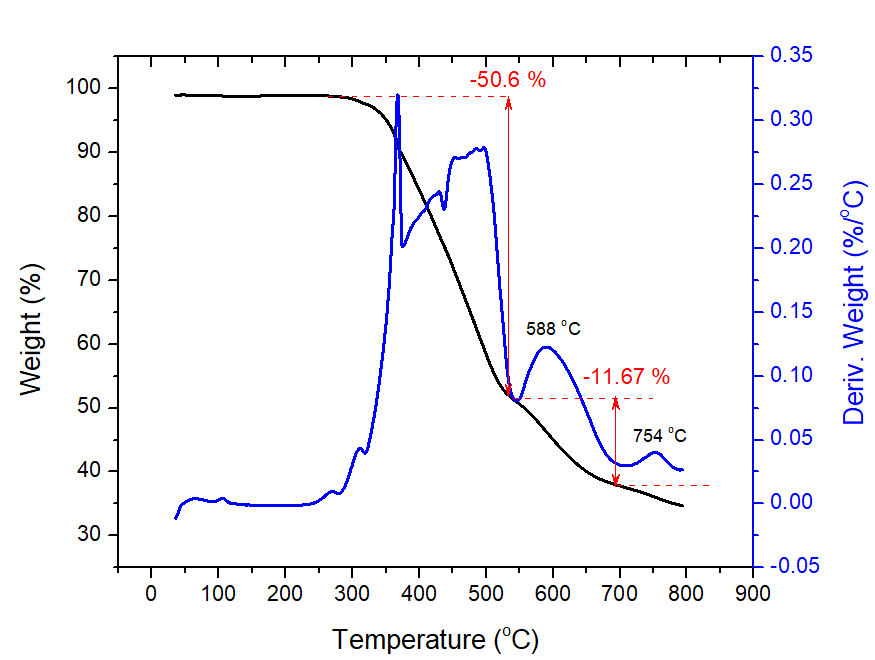


**Figure S2.** TGA-DTA plot of Co(2,2’-bipy)Cl_2_.


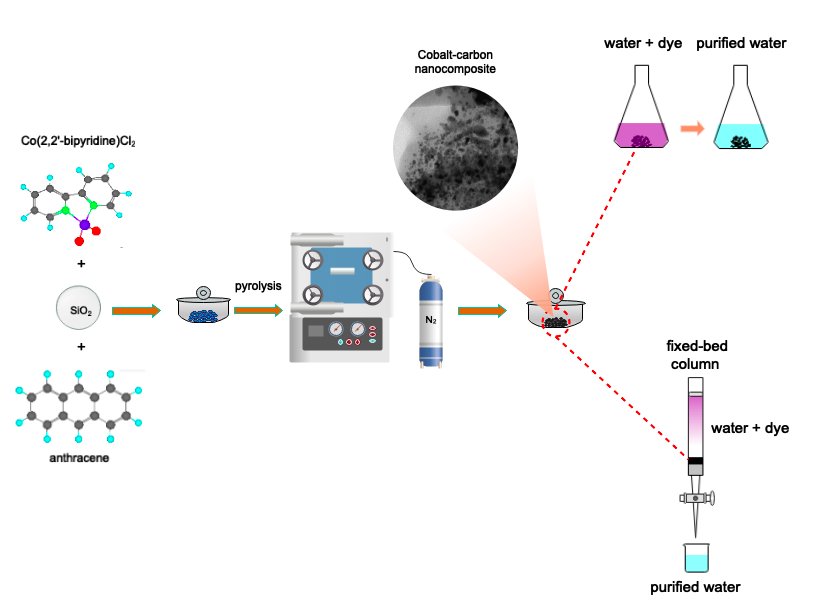


**Figure S3.** An illustrative diagram of cobalt-carbon nanocomposite and its application in Basic Violet 3 dye removal from aqueous solution using batch and column methods.


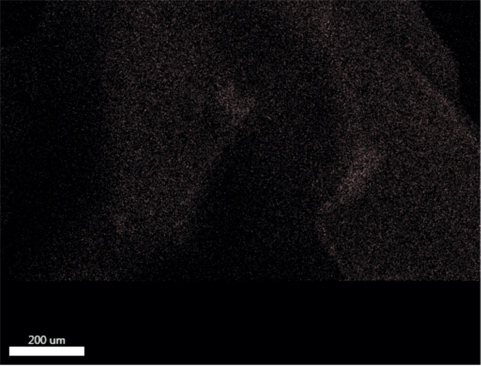

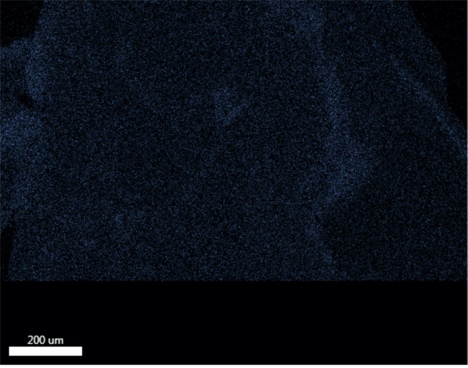

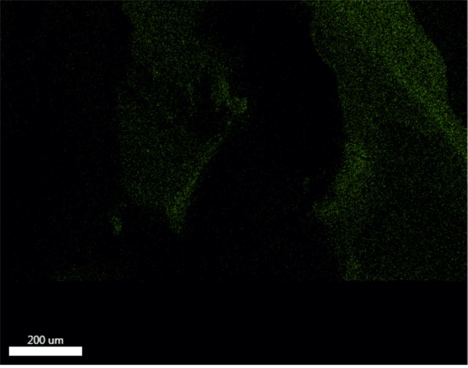

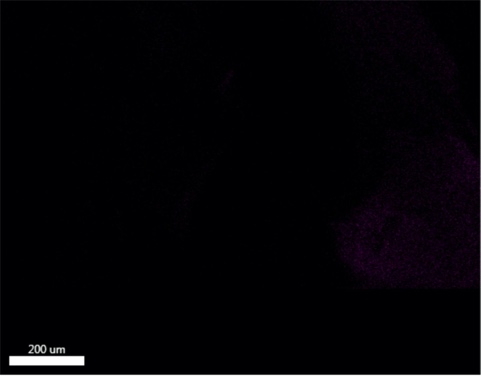

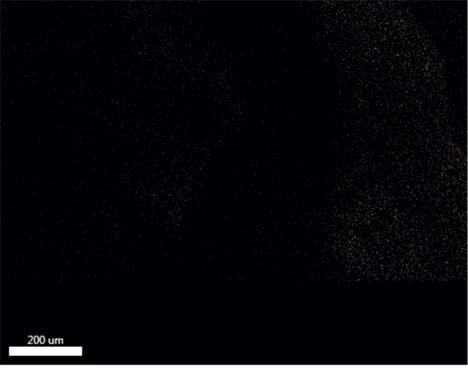

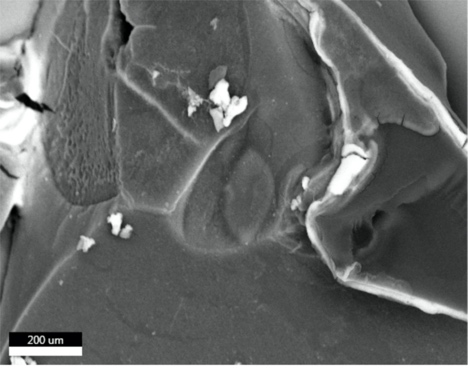

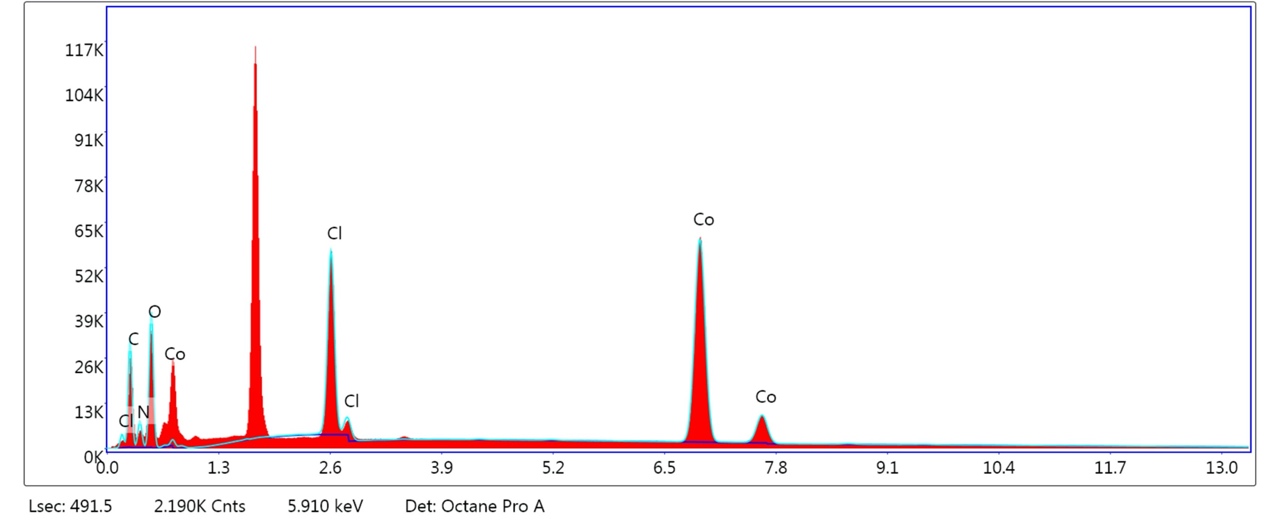


**Cl**

**C**

**Co**

**O**

**N**

| Element | Weight % | Atomic % | Net Int. | Error % | Kratio | Z | A | F |  |  |  |
| --- | --- | --- | --- | --- | --- | --- | --- | --- | --- | --- | --- |
| C K | 36.22 | 51.90 | 337.30 | 8.68 | 0.0853 | 1.0913 | 0.2504 | 1.0000 | |  | |
| N K | 10.53 | 13.27 | 88.70 | 10.36 | 0.0131 | 1.0672 | 0.1166 | 1.0000 | |  | |
| O K | 21.49 | 15.34 | 489.90 | 9.77 | 0.0386 | 1.0460 | 0.1341 | 1.0000 | |  | |
| ClK | 6.37 | 3.17 | 1090.90 | 2.95 | 0.0480 | 0.8895 | 0.8322 | 1.0179 | |  | |
| CoK | 24.38 | 17.30 | 1819.20 | 1.99 | 0.2023 | 0.7869 | 1.0345 | 1.0194 | |  | |

**Figure S4.** EDX spectrum and EDX elemental mapping of CoCNC **(2)**.


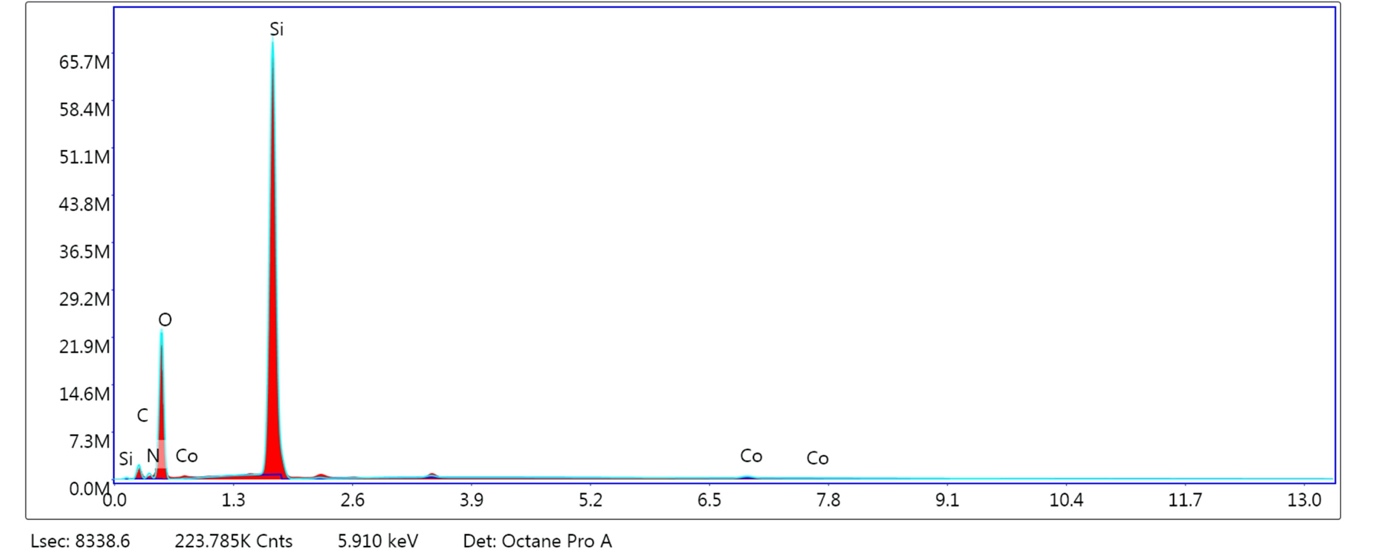

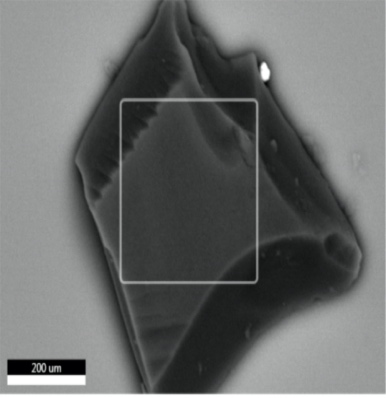

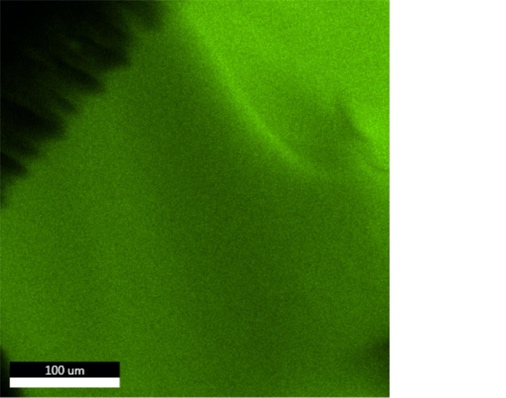

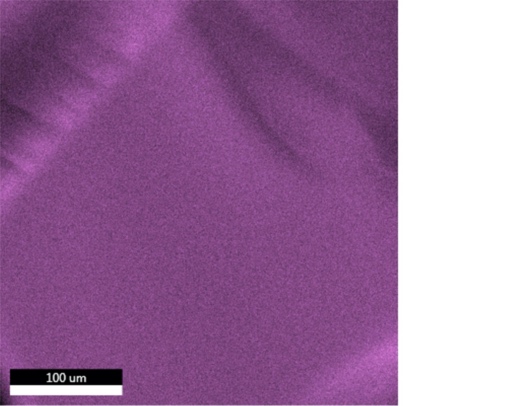

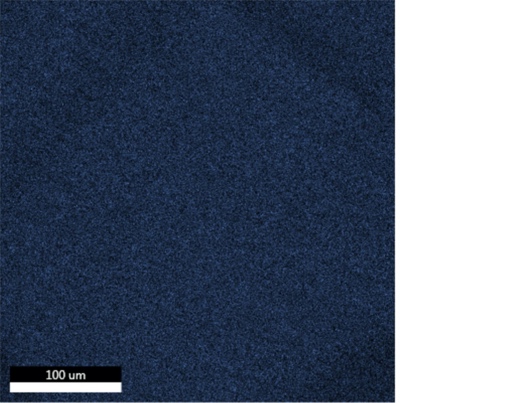

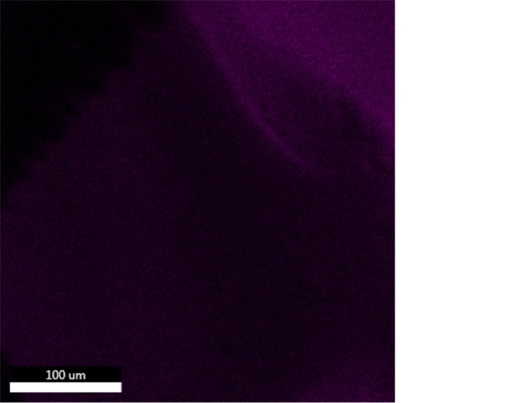

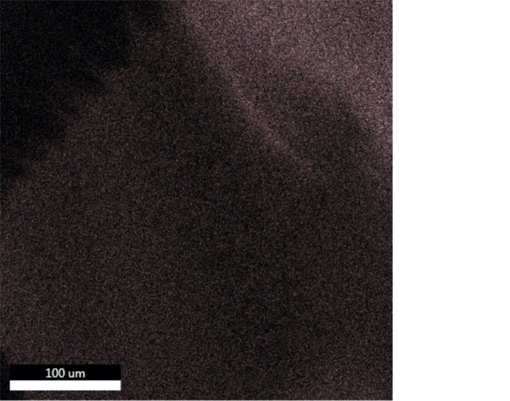


**Co**

**C**

**Si**

**O**

**N**

| Element | Weight % | Atomic % | Net Int. | Error % | Kratio | Z | A | F |  |
| --- | --- | --- | --- | --- | --- | --- | --- | --- | --- |
| C K | 34.50 | 40.95 | 1333.70 | 9.28 | 0.0259 | 1.0647 | 0.1678 | 1.0000 | |
| N K | 4.12 | 5.11 | 557.90 | 9.50 | 0.0063 | 1.0403 | 0.1476 | 1.0000 | |
| O K | 21.03 | 25.36 | 1144.40 | 8.96 | 0.1037 | 1.0190 | 0.1994 | 1.0000 | |
| SiK | 30.81 | 16.42 | 68756.30 | 4.39 | 0.1835 | 0.9262 | 0.6638 | 1.0012 | |
| CoK | 10.54 | 12.16 | 555.10 | 2.57 | 0.0047 | 0.7606 | 1.0294 | 1.1299 | |

**Figure S5.** EDX spectrum and EDX elemental mapping of CoCNC@SiO_2_ **(3)**.

**Table S2.** Elemental analysis results of CoCNC **(2)** and CoCNC@SiO_2_ **(3)**.

| Adsorbent | % of N | % of C | % of H |
| --- | --- | --- | --- |
| CoCNC **(2)** | 8.316 | 41.924 | 0.1184 |
| CoCNC@SiO_2_ **(3)** | 5.36 | 31.904 | 0.0182 |


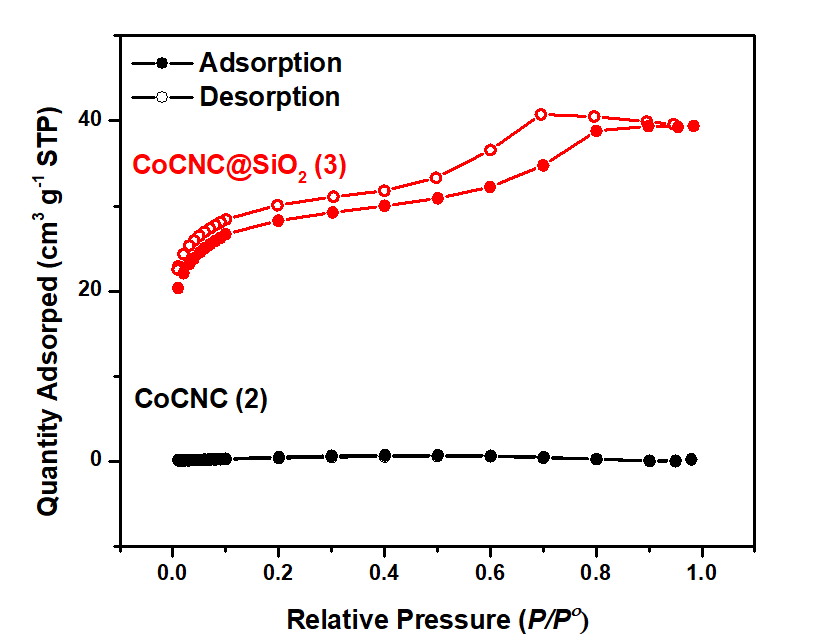


**Figure S6.** Nitrogen adsorption/desorption isotherm at 77K of CoCNC **(2)** and CoCNC@SiO_2_ **(3)**.


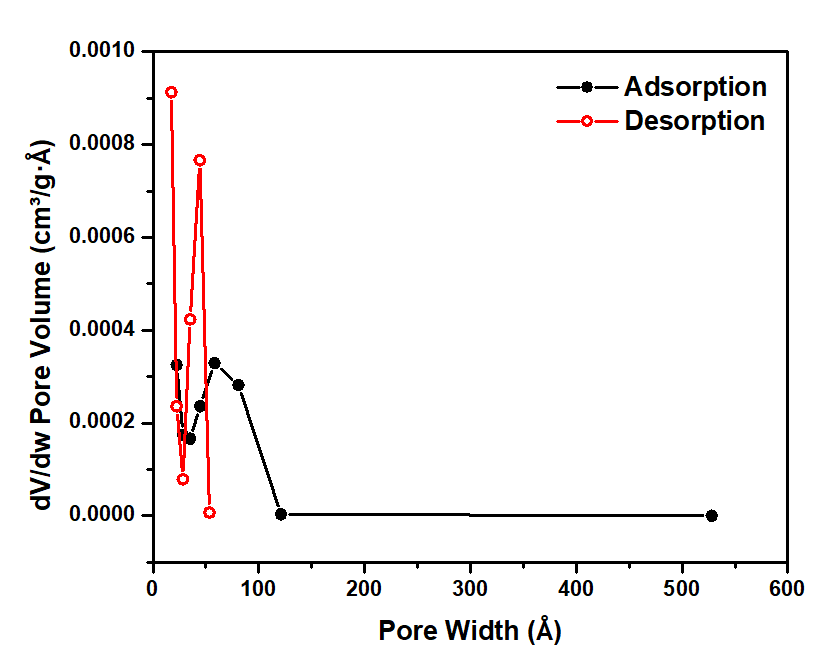


**Figure S7.** Barrett-Joyner-Halenda (BJH) pore-size distribution CoCNC@SiO_2_ **(3)**.


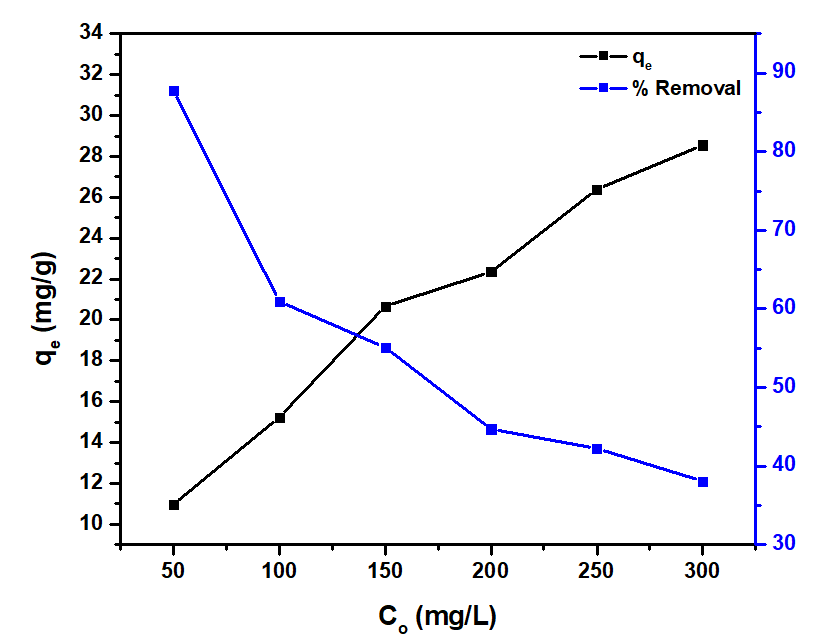


**Figure S8.** Effect of initial dye concentration on the amount of Basic Violet 3 adsorbed onto CoCNC@SiO_2_ **(3)** from aqueous solution at 25 ^0^C.

**
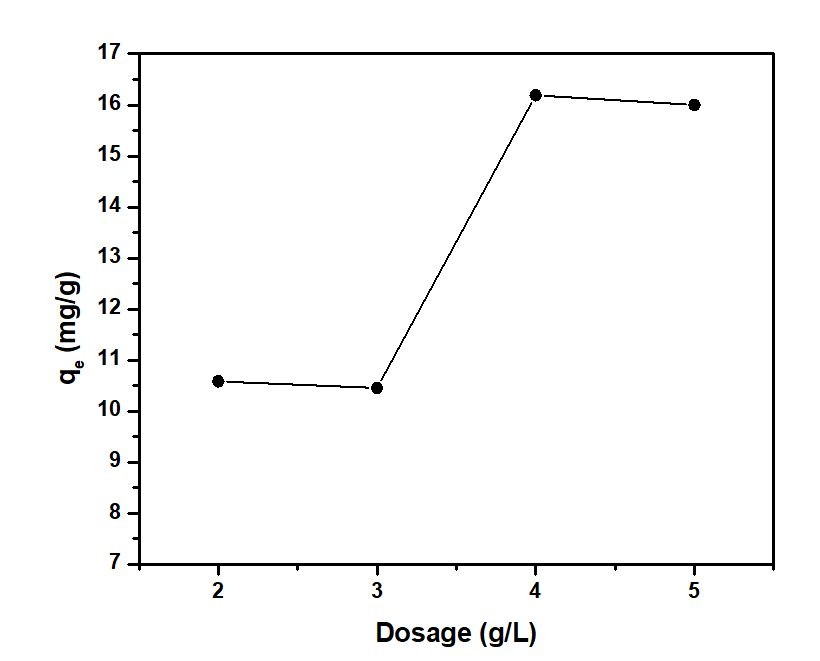
**

**Figure S9.** Effect of adsorbent dosage on the adsorption of Basic Violet 3 on CoCNC@SiO_2_ **(3)**.


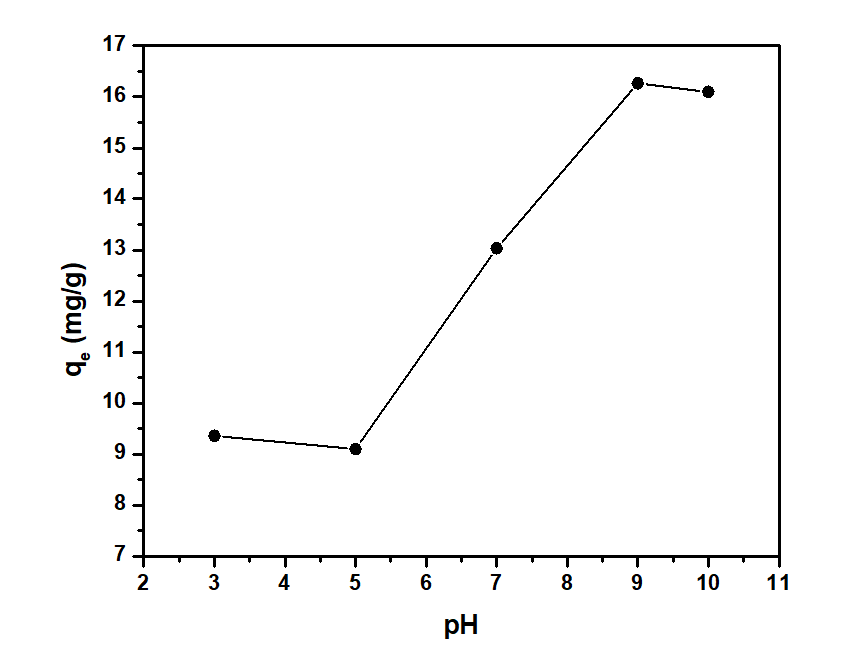


**Figure S10.** Effect of pH on the adsorption of Basic Violet 3 on CoCNC@SiO_2_ **(3)**.


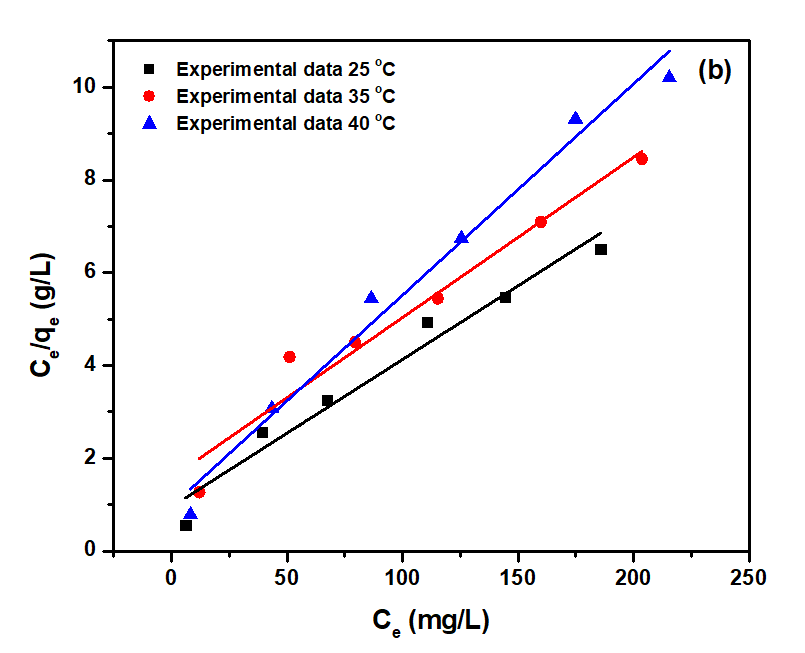
**
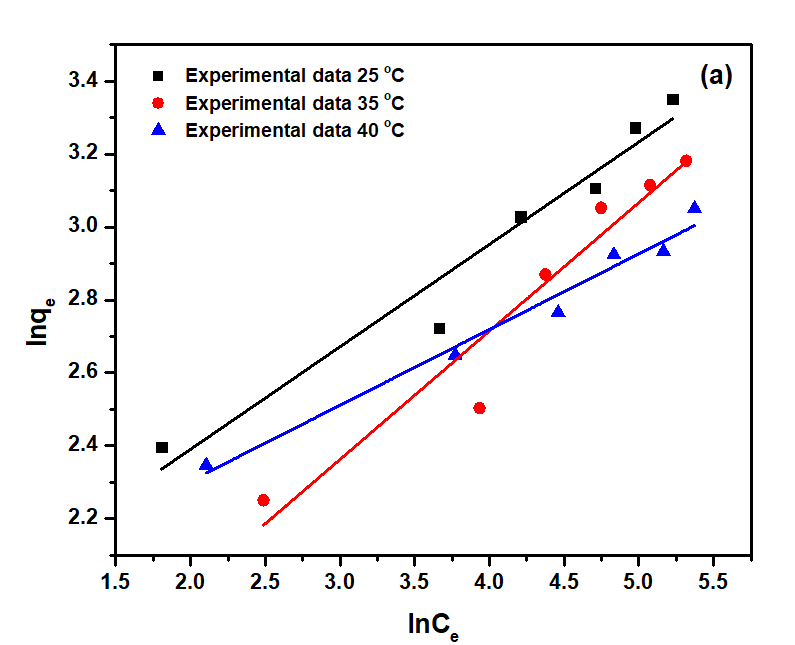
**

**Figure S11.** (a) Linear fit to Freundlich model. (b) Linear fit to Langmuir model for the adsorption of Basic Violet 3 onto CoCNC@SiO_2_ **(3)**.


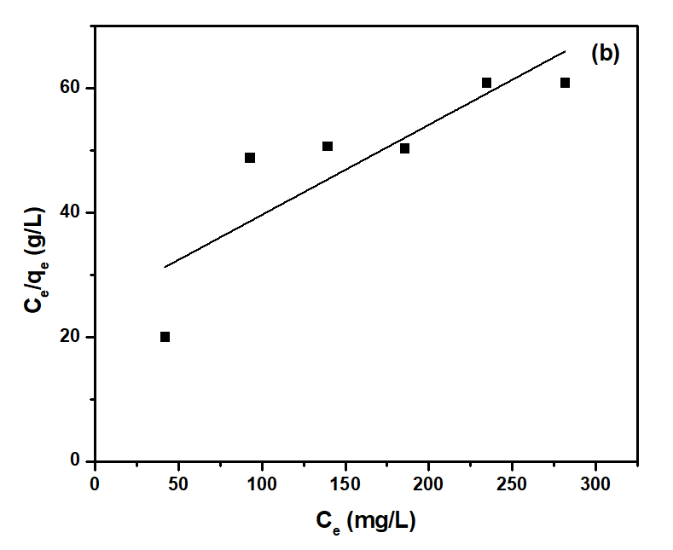

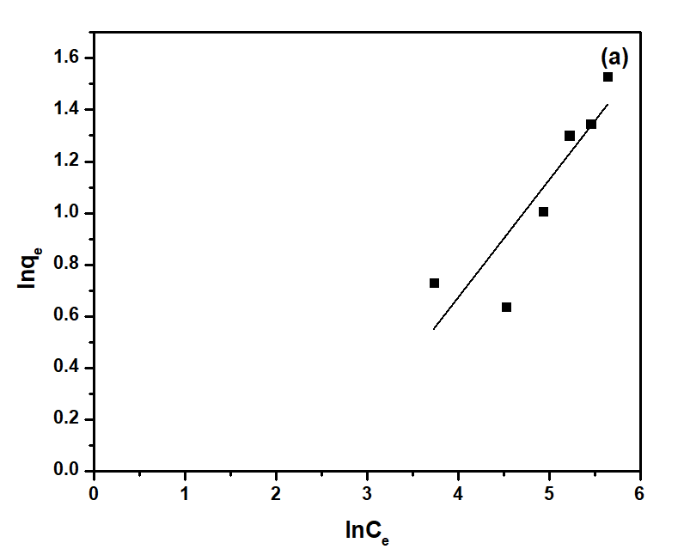


**Figure S12.** (a) Linear fit to Freundlich model. (b) Linear fit to Langmuir model for the adsorption of Basic Violet 3 onto CoCNC **(2)**.


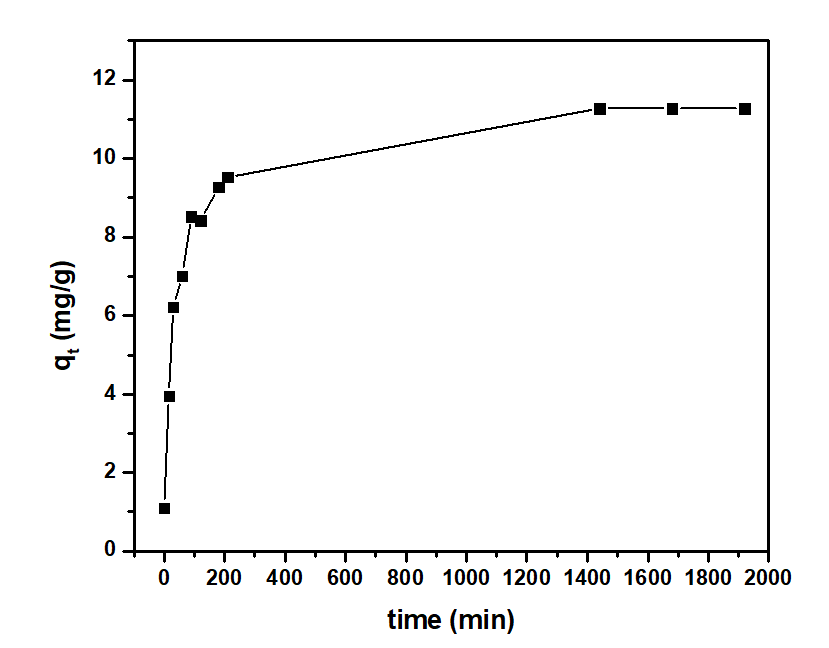


**Figure S13.** Effect of contact time on the adsorption capacity of CoCNC@SiO_2_ **(3)** at 25 ^o^C.


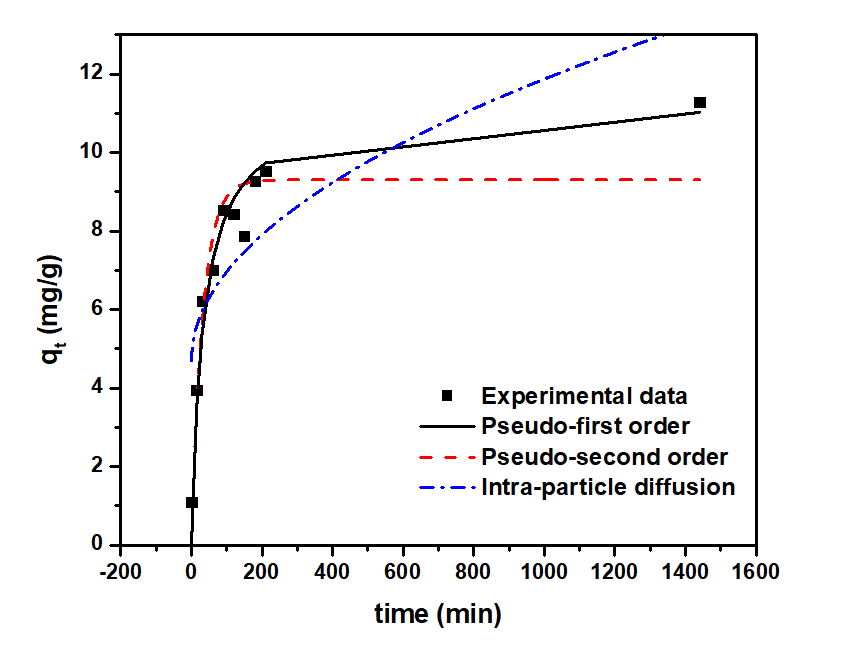


**Figure S14.** Non linear adsorption kinetics of CV adsorption onto CoCNC@SiO_2_ **(3)** at 25 ^o^C.


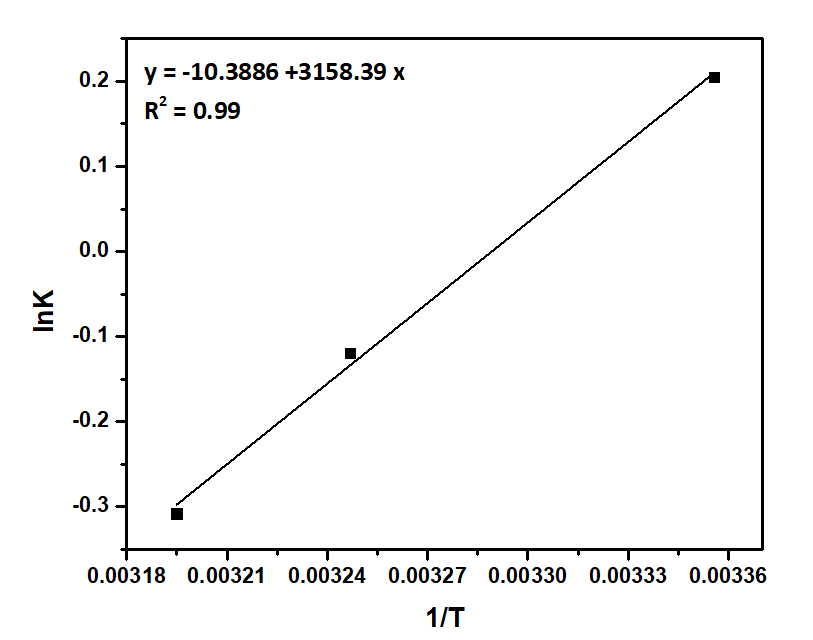
**Figure S15.** lnK *vs* 1/T plot for CoCNC@SiO_2_ **(3)**.


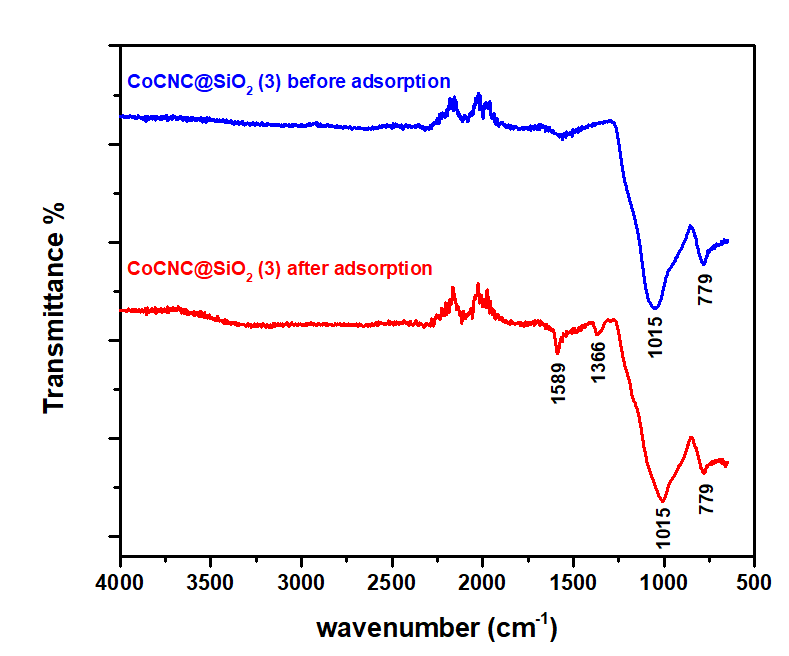


**Figure S16.** FTIR spectrum of CoCNC@SiO_2_ **(3)** before and after adsorption.

References

1. Preda, N. *et al.* The intercalation of pbi_2_ with 2,2 ’-bipyridine evidenced by photoluminescence. FT-IR and Raman spectroscopy. *Rom. J. Phys.* **54**, 667–675 (2009).
